# Size-Related Changes in Foot Impact Mechanics in Hoofed Mammals

**DOI:** 10.1371/journal.pone.0054784

**Published:** 2013-01-30

**Authors:** Sharon Elaine Warner, Phillip Pickering, Olga Panagiotopoulou, Thilo Pfau, Lei Ren, John Richard Hutchinson

**Affiliations:** 1 Structure and Motion Laboratory, Comparative Biomedical Sciences, The Royal Veterinary College, Hatfield, Hertfordshire, United Kingdom; 2 Veterinary Clinical Sciences, The Royal Veterinary College, Hatfield, Hertfordshire, United Kingdom; 3 School of Mechanical, Aerospace and Civil Engineering, The University of Manchester, Manchester, Greater Manchester, United Kingdom; University of Missouri, United States of America

## Abstract

Foot-ground impact is mechanically challenging for all animals, but how do large animals mitigate increased mass during foot impact? We hypothesized that impact force amplitude scales according to isometry in animals of increasing size through allometric scaling of related impact parameters. To test this, we measured limb kinetics and kinematics in 11 species of hoofed mammals ranging from 18–3157 kg body mass. We found impact force amplitude to be maintained proportional to size in hoofed mammals, but that other features of foot impact exhibit differential scaling patterns depending on the limb; forelimb parameters typically exhibit higher intercepts with lower scaling exponents than hind limb parameters. Our explorations of the size-related consequences of foot impact advance understanding of how body size influences limb morphology and function, foot design and locomotor behaviour.

## Introduction

Mitigating the mechanical consequences of foot-ground impact is critically important to musculoskeletal tissue health. The repeated, cyclical applications of transient forces have been implicated in fatigue accumulation leading to tissue failure [1]–[4]; it is the temporal nature of impact (i.e. rate and frequency of force application) that is thought to make loading during this phase particularly destructive [2], [3]. As terrestrial cursors [5], hoofed mammals may be particularly vulnerable to fatigue damage because their relatively stiff, erect limbs coupled with presumably limited foot compliance (due to their small, rigid hooves) have to withstand long daily travel distances and rapid speeds. Although no real data exist to demonstrate the importance of fatigue damage in non-domesticated species, we wondered how large hoofed mammals are able to cope with highly repetitive impact loads particularly as ungulates span such an impressive body size range.

The scaling of geometric volumes suggest that impact loading poses a greater challenge to larger, heavier ungulates because forces (inertial and gravitational) are proportional to mass. Previous work however, has shown that peak bone stresses are maintained with size, through changes in limb posture and duty factor, by reducing locomotor performance and in very large animals, by increasing bone robusticity [6], [7]. Furthermore, the frequency at which these forces (and thus stresses) are applied is reduced [8], [9]. We therefore consider impact force amplitude alongside other features of foot impact in order to explore how increased body mass is mitigated during impact loading. We use the geometric similarity model as the basis of our hypotheses because many aspects of the model are supported over a wide range of species [6]–[11]. With insufficient evidence to indicate that larger ungulates are more susceptible to mechanical injury, we speculate that allometric relationships may exist to ensure that impact mechanics remain within tolerable limits.

At foot impact, the magnitude of force experienced by the limb is determined by the mass and acceleration of the limb. Whereas segmental mass is pre-determined by morphology (increasing isometrically in geometrically similar animals), effective foot mass (M*_eff_*); i.e. the amount of limb mass than collides with the ground prior to the limb being loaded with body mass (see Methods section [12]); may vary with neuromuscular control of limb dynamics. Although geometric similarity suggests that muscle force is proportional to physiological cross sectional area (expected to scale ∼M_b_^0.67^), changes in limb posture ensure that peak muscle stresses remain constant with increasing body mass [6]. Previous work by More *et al*. [13] suggests that despite having similar muscle force producing capacity, large and small animals may differ in their ability to respond to external stimuli, particularly to rapid events like foot impact. We expect M*_eff_* to remain isometric (scaling ∼M_b_^ 0.84^), with forelimb M*_eff_* being greater than hind limb M*_eff_* [14]. (For more information on how this value (0.84) was derived, see equation 1 and the text in the following paragraphs explaining how impact impulse, velocity and duration are expected to scale).

Extending the time period over which a collision takes place decreases acceleration and therefore impact force magnitude [15]. In the case of foot impact in humans, the latency period of muscle prevents the body from actively extending impact duration via changing limb geometry or limb stiffness [16]. Although passive damping is likely to be limited by foot morphology (rigid hoof, coupled with a small digital cushion), ungulates may be able to use the relative movement of the digits, a sequential landing pattern and foot slip to prolong impact. We speculate that these features are unlikely to change with body mass; however, stance duration has previously been shown to increase with body mass [6], [10]. On that basis, we expect impact duration to scale with positive allometry (scaling with a slope higher than M_b_^0.17^ ) in both the fore- and hind limbs. This value is derived from [17], who proposed that time related variables scale proportionally to the square root of length variables.

As a consequence of longer impact duration, we expect loading rate to scale with negative allometry (scaling with a slope lower than M_b_^0.83^; derived from loading rate or force divided by time; i.e. dividing M_b_^1.00^ by M_b_^0.17^). Considering that the forelimb functions primarily to brake centre of mass (CoM) motion in most mammalian quadrupeds [11], we anticipate that the forelimb will experience a higher loading rate than the hind limb [18]. Additionally, we expect impact impulse (i.e. the integral of force and time) to remain isometric (scaling ∼M_b_^ 1.17^). Considering that the hind limbs function primarily to propel the CoM in most mammals [11], we predict that the hind limbs will experience greater impact impulses than the forelimbs. While segment length scales isometrically in geometrically similar animals, limb angle at impact seems to scale lower than what isometry predicts [19]. On that basis, we expect (horizontal) impact velocity to scale with negative allometry (scaling with a slope lower than M_b_^0.16^), with the (propulsive) hind limbs impacting at faster velocities than the forelimbs [18], [20]. The expected value of 0.16 for geometric similarity is derived from dividing distance (M_b_^0.33^) by time (M_b_^0.17^)_._

**Figure 1:**
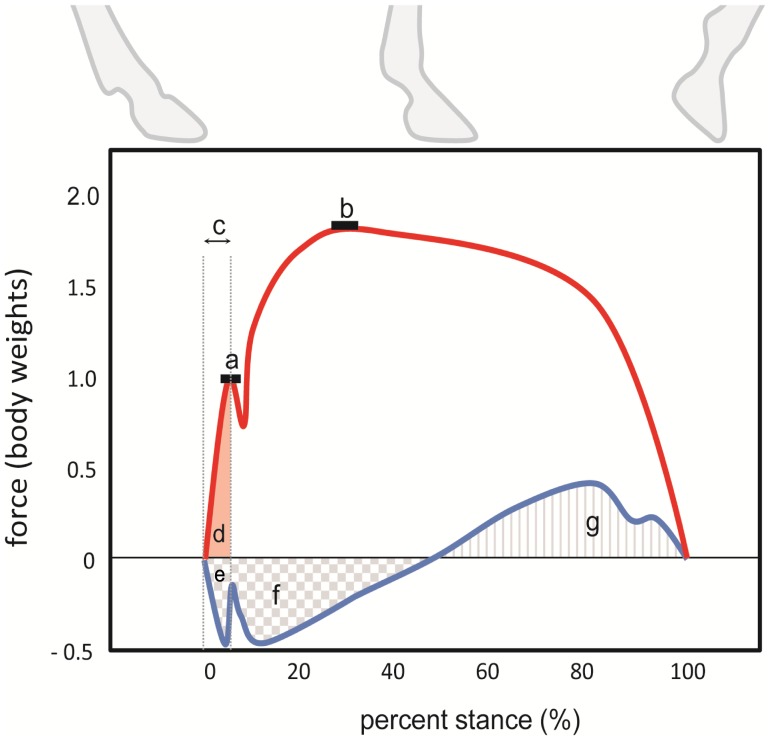
Schematic diagram of a single limb’s 2D force trace showing impact parameters of interest. a) peak vertical impact force amplitude; b) peak vertical ground reaction force (GRF) amplitude; c) impact duration; d) vertical horizontal impact impulse and e) horizontal impact impulse; f) total decelerative impulse over the entire stance; g) total accelerative impulse over the entire stance.

Here, for the first time in a broad comparative context we determine how features of foot impact scale in hoofed mammals spanning over two orders of magnitude of adult body mass. Using the geometric similarity model, we hypothesise that impact force amplitude (Fig. 1a) will remain isometric with increasing size (scaling ∼M_b_^1.00^), through allometric changes in impact velocity and impact duration (Fig. 1c). Larger animals are likely to experience lower impact velocities and longer impact durations, countering the increased mass associated with their larger size. Consequently, loading rate is likely to scale allometrically while M*_eff_* and impact impulse (Fig. 1d and 1e) remain isometric. Furthermore, due to differential limb function as noted above, there are likely to be disparities between how fore- and hindlimb impact dynamics scale [21].

## Results

**Figure 2:**
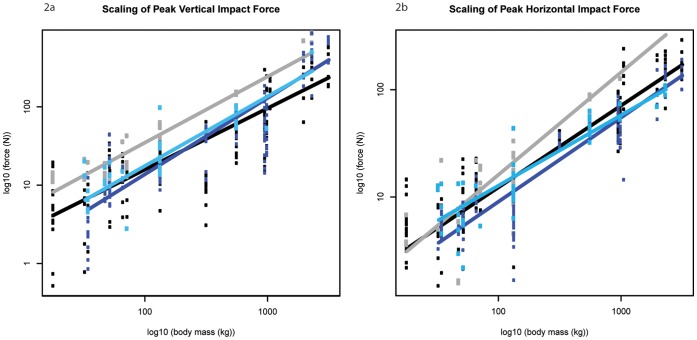
Scaling outcomes for peak impact force amplitude. a) peak vertical impact force amplitude; b) peak horizontal impact force amplitude. Black markers denote forelimb walk data; grey markers denote forelimb slow run data; dark blue markers denote hind limb walk data; light blue markers denote hind limb slow run data. The correspondingly coloured trend lines represent the scaling outcome generated by the LMM analysis.

**Figure 3:**
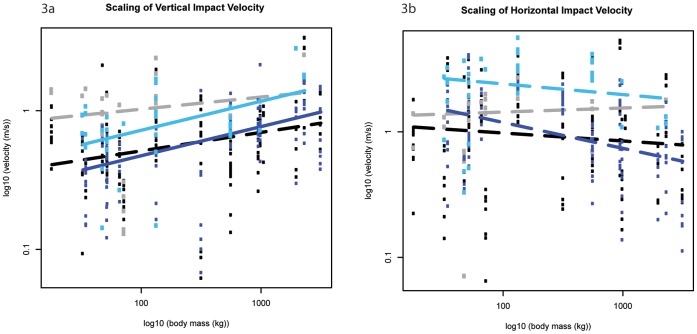
Scaling outcomes for impact velocity. a) vertical impact velocity; b) horizontal impact velocity. Black markers denote forelimb walk data; grey markers denote forelimb slow run data; dark blue markers denote hind limb walk data; light blue markers denote hind limb slow run data. The correspondingly coloured trend lines represent the scaling outcome generated by the LMM analysis. Dashed lines show non-significant scaling outcomes, i.e. the slope is not different from a slope of zero.

**Figure 4:**
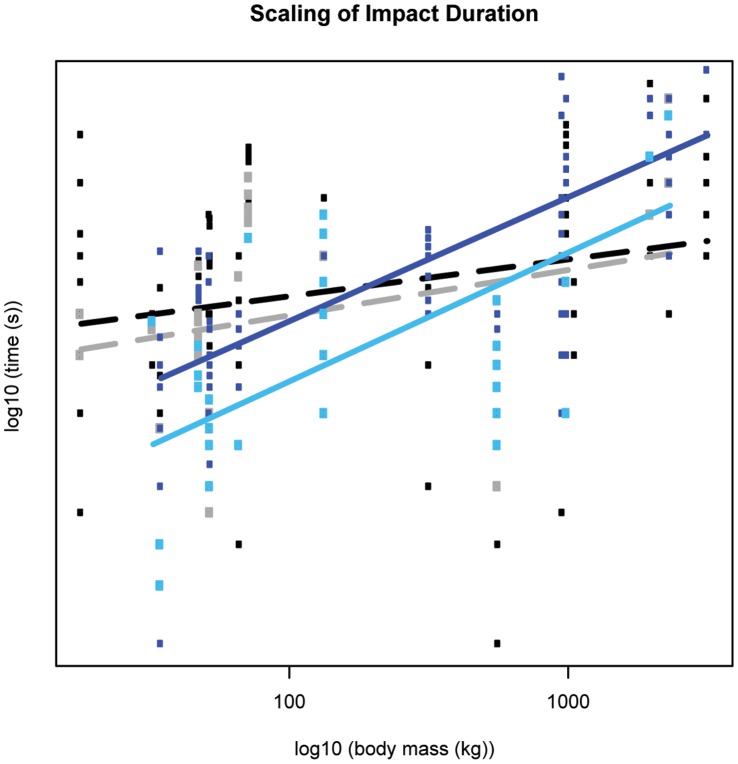
Scaling outcome for impact duration. Black markers denote forelimb walk data; grey markers denote forelimb slow run data; dark blue markers denote hind limb walk data; light blue markers denote hind limb slow run data. The correspondingly coloured trendlines represent the scaling outcome generated by the LMM analysis. Dashed lines show non-significant scaling outcomes, i.e. the slope is not different from a slope of zero.

**Figure 5:**
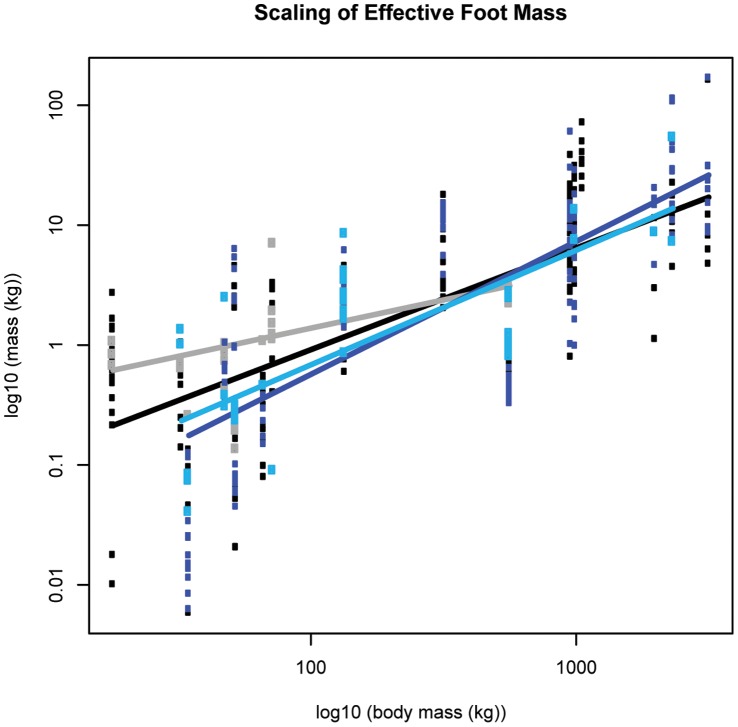
Scaling outcomes for effective foot mass (M*_eff_*). Black markers denote forelimb walk data; grey markers denote forelimb slow run data; dark blue markers denote hind limb walk data; light blue markers denote hind limb slow run data. The correspondingly coloured trendlines represent the scaling outcome generated by the LMM analysis.

**Figure 6:**
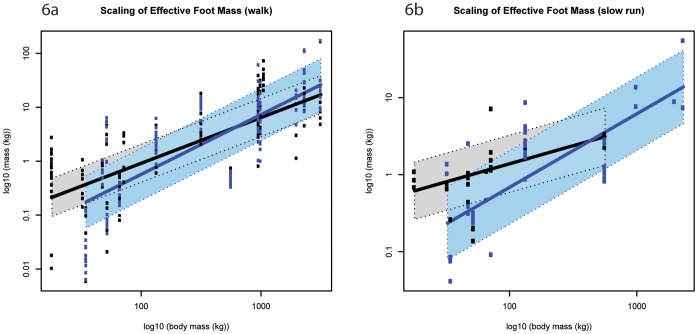
Standard errors of model fit (M*_eff_*). a) walk; b) slow run. Black markers denote forelimb data; blue markers denote hind limb data. The corresponding shaded areas show two standard errors from the fitted model. Although the intersection suggests that smaller species (below ∼750 kg M_b_) have greater forelimb M*_eff_,* whereas larger species appeared to have greater hind limb M*_eff_*, the standard errors associated with model fitting mean these limb differences are not statistically significant.

**Figure 7:**
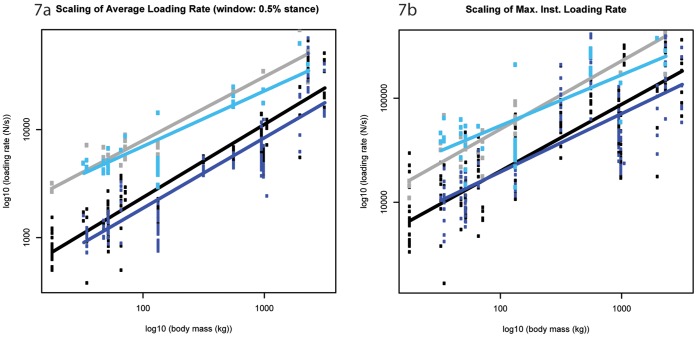
Scaling outcomes for loading rate. a) maximum average loading rate (calculated over 0.5% rolling window throughout the impact period); b) maximum instantaneous rate of force application. Black markers denote forelimb walk data; grey markers denote forelimb slow run data; dark blue markers denote hind limb walk data; light blue markers denote hind limb slow run data. The correspondingly coloured trendlines represent the scaling outcome generated by the LMM analysis.

**Figure 8:**
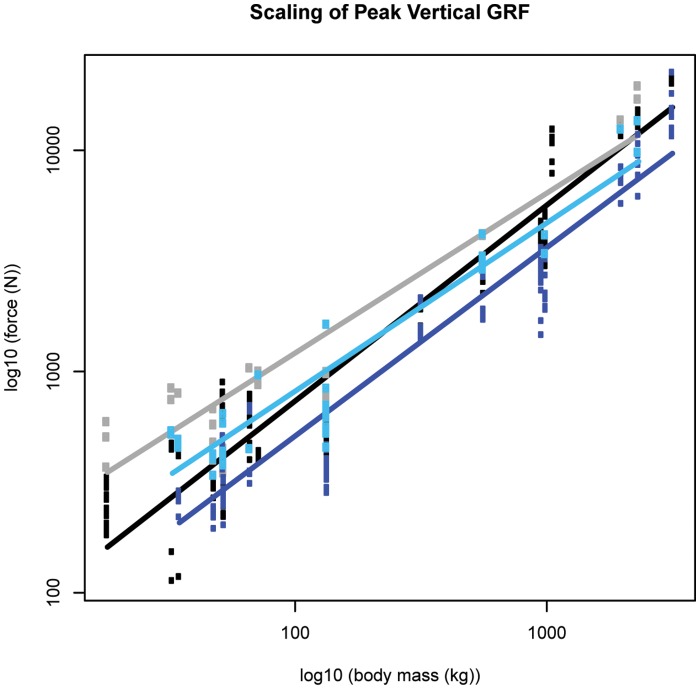
Scaling outcome for peak vertical ground reaction force (GRF). Black markers denote forelimb walk data; grey markers denote forelimb slow run data; dark blue markers denote hind limb walk data; light blue markers denote hind limb slow run data. The correspondingly coloured trendlines represent the scaling outcome generated by the LMM analysis.

**Figure 9:**
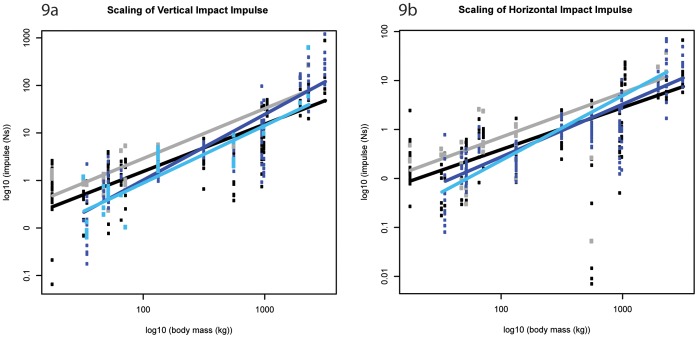
Scaling outcomes for impact impulse. a) vertical impact impulse; b) horizontal impact impulse. Black markers denote forelimb walk data; grey markers denote forelimb slow run data; dark blue markers denote hind limb walk data; light blue markers denote hind limb slow run data. The correspondingly coloured trendlines represent the scaling outcome generated by the LMM analysis.

**Figure 10:**
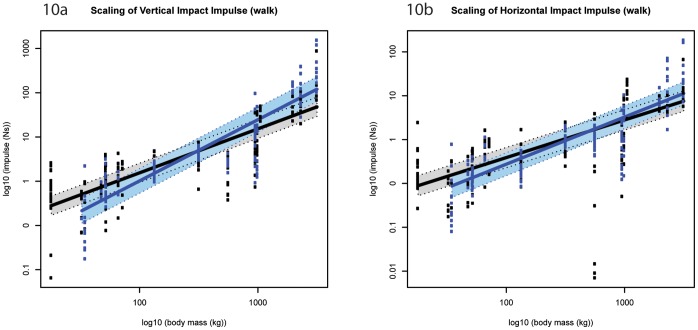
Standard errors of model fit (impact impulse). a) vertical impact impulse (walk); b) horizontal impact impulse (walk). Black markers denote forelimb walk data; blue markers denote hind limb walk data. The corresponding shaded areas show two standard errors from the fitted model. Although the intersection suggests that smaller species (below ∼750 kg M_b_) have greater forelimb impact impulses, whereas larger species appeared to have greater hind limb impact impulses, the standard errors associated with model fitting mean these limb differences are not statistically significant.

**Figure 11:**
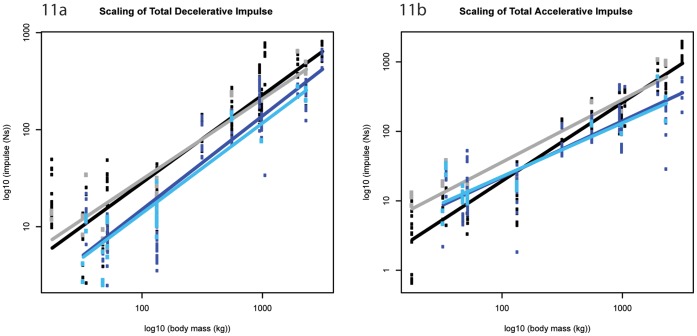
Scaling outcomes for total impulses. a) total decelerative impulse; b) total accelerative impulse. Black markers denote forelimb walk data; grey markers denote forelimb slow run data; dark blue markers denote hind limb walk data; light blue markers denote hind limb slow run data. The correspondingly coloured trendlines represent the scaling outcome generated by the LMM analysis.

**Figure 12:**
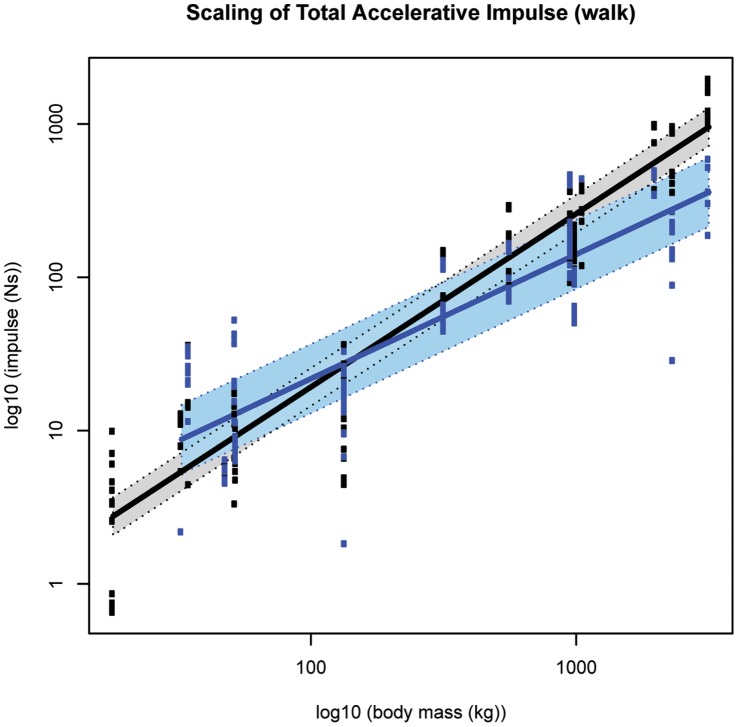
Standard errors of model fit (total accelerative impulse). Black markers denote forelimb walk data; blue markers denote hind limb walk data. The corresponding shaded areas show two standard errors from the fitted model. Although the intersection suggests that smaller species (below ∼300 kg M_b_) have greater hindlimb accelerative impulses, whereas larger species appeared to have greater forelimb impact impulses, the standard errors associated with model fitting mean these limb differences are not statistically significant in all but extreme body sizes.

Here we present our results for each parameter of impact dynamics; the exponent predicted by isometry is in parentheses after each subheading. Our LMM (linear mixed effect model) analysis (see Methods) provided the mean scaling exponents ± standard errors. Note that this approach differs from standard bivariate scaling regression, but produces comparable patterns that allow more statistically rigorous inferences about scaling to be made. Detailed results are shown in Figures 2, 3, 4, 5, 6, 7, 8, 9, 10, 11, and 12, Tables 1 and 2, and also Tables S1–S27. Some apparent patterns of scaling were non-significant trends, but still strikingly different for the fore- vs. hind limbs. Therefore, in addition to explicitly presenting significant scaling patterns here, we denote any non-significant pattern that is of potential biological significance as a “trend”, with the hope that this clarity inspires future scaling studies to more unambiguously test their significance.

**Table 1:** Kinetic impact dynamic parameters.

			exponent	std. error	p value (zero)	p value (isometry)	conclusion	trend	intercept	t statistic	df	n species	n indiv.	n impacts
peak vertical impact force amplitude (M^1.00^)	Forelimb	walk	0.78*	0.07	<0.01	0.01	negative allometry		0.63	−3.14	15	11	17	202
		slow run	0.84	0.1	<0.01	0.12	isometry	negative allometry	0.85	−1.60	28	7	7	36
	Hind limb	walk	0.97	0.12	<0.01	0.83	isometry	negative allometry	0.19	−0.25	11	9	13	153
		slow run	0.89	0.18	<0.01	0.54	isometry	negative allometry	0.47	−0.61	35	7	7	43
peak horizontal impact force amplitude (M^1.00^)	Forelimb	walk	0.76*	0.05	<0.01	<0.01	negative allometry		0.56	−4.80	15	11	17	204
		slow run	0.96	0.12	<0.01	0.71	isometry	negative allometry	0.30	−0.33	28	7	8	37
	Hind limb	walk	0.78*	0.06	<0.01	<0.01	negative allometry		0.39	−3.67	13	9	15	151
		slow run	0.66*	0.06	<0.01	<0.01	negative allometry		0.79	−5.67	35	7	7	43
M*_eff_* (M^0.84^)	Forelimb	walk	0.85	0.12	<0.01	0.96	isometry	positive allometry	−1.73	−1.25	15	11	17	199
		slow run	0.47	0.19	0.02	0.06	isometry	negative allometry	−0.80	−2.79	25	7	5	32
	Hind limb	walk	1.11	0.24	<0.01	0.29	isometry	positive allometry	−2.46	0.46	11	9	13	150
		slow run	0.96	0.24	<0.01	0.63	isometry	positive allometry	−2.08	−0.17	34	7	6	42
vertical impact impulse (M^1.17^)	Forelimb	walk	0.99	0.11	<0.01	0.13	isometry	negative allometry	−1.80	−0.09	15	11	17	199
		slow run	1.05	0.22	<0.01	0.59	isometry	negative allometry	−1.64	0.23	28	7	7	36
	Hind limb	walk	1.38	0.14	<0.01	0.15	isometry	positive allometry	−2.76	2.71	12	9	14	153
		slow run	1.21	0.22	<0.01	0.86	isometry	positive allometry	−2.47	0.95	34	7	6	42
(abs) horizontal impact impulse (M^1.17^)	Forelimb	walk	0.86*	0.12	<0.01	0.02	negative allometry		−2.13	−1.17	15	11	17	194
		slow run	0.91	0.23	<0.01	0.25	isometry	negative allometry	−1.97	−0.39	25	7	6	33
	Hind limb	walk	1.08	0.13	<0.01	0.49	isometry	negative allometry	−2.71	0.62	11	10	13	152
		slow run	1.32	0.14	<0.01	0.29	isometry	positive allometry	−3.28	2.29	35	7	6	43
loading rate (0.5% stance window) (M^0.83^)	Forelimb	walk	0.68	0.05	<0.01	0.01	negative allometry		5.02	−6.40	15	11	17	204
		slow run	0.59	0.06	<0.01	<0.01	negative allometry		5.72	−6.83	28	7	8	37
	Hind limb	walk	0.65	0.07	<0.01	0.02	negative allometry		4.97	−5.00	13	9	15	152
		slow run	0.51	0.08	<0.01	<0.01	negative allometry		5.82	−6.13	35	7	7	43
max. inst. loading rate (M^0.83^)	Forelimb	walk	0.64*	0.08	<0.01	0.04	negative allometry		3.02	−4.50	15	11	17	204
		slow run	0.66	0.09	<0.01	0.06	isometry	negative allometry	3.39	−3.78	28	7	8	37
	Hind limb	walk	0.56*	0.11	<0.01	0.03	negative allometry		3.17	−4.00	13	9	15	152
		slow run	0.49*	0.12	<0.01	0.01	negative allometry		3.76	−4.25	35	7	7	43
peak vertical GRF (M^1.00^)	Forelimb	walk	0.89	0.06	<0.01	0.06	isometry	negative allometry	1.10	−1.83	15	11	17	202
		slow run	0.72*	0.10	<0.01	0.01	negative allometry		1.64	−2.80	28	7	7	36
	Hind limb	walk	0.85*	0.06	<0.01	0.03	negative allometry		1.00	−2.50	11	9	13	153
		slow run	0.76*	0.08	<0.01	<0.01	negative allometry		1.39	−3.00	35	7	7	43
total decelerative impulse (M^1.17^)	Forelimb	walk	0.90	0.09	<0.01	0.01	negative allometry		−0.35	10.08	13	11	15	183
		slow run	0.83	0.13	<0.01	0.02	negative allometry		−0.17	6.28	23	7	7	31
	Hind limb	walk	0.96	0.10	<0.01	0.06	isometry	negative allometry	−0.75	9.86	12	10	14	146
		slow run	0.93	0.14	<0.01	0.08	isometry	negative allometry	−0.72	6.86	34	6	6	41
total accelerative impulse (M^1.17^)	Forelimb	walk	1.13	0.06	<0.01	0.54	isometry		−0.98	18.33	13	11	15	183
		slow run	0.90	0.13	<0.01	0.05	negative allometry		−0.24	6.95	23	7	7	31
	Hind limb	walk	0.81	0.11	<0.01	0.01	negative allometry		−0.28	7.20	12	10	14	146
		slow run	0.77	0.14	<0.01	0.01	negative allometry		−0.19	5.43	34	6	6	41

Shown: scaling exponent (from LMM analysis), exponent standard error, p value determining if the slope of the data is significantly different from zero, p value determining if the slope of the data is significantly different from the isometric prediction, scaling analysis conclusion, scaling trend (if conclusion is not significantly different from isometry), intercept, t statistic (observed slope vs. slope predicted by isometry), degrees of freedom, number of species, number of individuals and number of impacts (i.e. instances of foot impact analysed) per impact variable. The exponent b is for the equation: log y = b log(M_b_)+log(a); where b is the slope (exponent); a is the elevation (y-intercept); and y is the impact parameter of interest. Asterisks denote an exponent that is significantly different from isometry.

**Table 2:** Kinematic impact dynamic parameters.

			exponent	std. error	p value (zero)	p value (isometry)	conclusion	trend	intercept	t statistic	df	n species	nindiv.	n impacts
vertical impact velocity (M^0.16^)	Forelimb	Walk	0.13	0.06	0.05	0.68	not significant		−0.56	−14.50	15	11	17	202
		Slow run	0.09	0.15	0.57	0.64	not significant		−0.16	−6.07	26	7	6	34
	Hind limb	Walk	0.21	0.05	<0.01	0.35	isometry	positive allometry	−0.74	−15.80	12	9	14	152
		Slow run	0.21	0.09	0.02	0.58	isometry	positive allometry	−0.56	−8.78	34	7	7	42
horizontal impact velocity (M^0.16^)	Forelimb	Walk	−0.06	0.11	0.57	0.06	not significant		0.12	−9.64	15	11	17	202
		Slow run	0.03	0.11	0.77	0.25	not significant		0.09	−8.82	26	7	6	34
	Hind limb	Walk	−0.21	0.10	0.07	<0.01	not significant		0.49	−12.10	11	9	13	151
		Slow run	−0.08	0.13	0.53	0.08	not significant		0.55	−8.31	34	7	5	42
impact duration (M^0.17^)	Forelimb	Walk	0.11	0.08	0.16	0.45	not significant		−1.87	−11.13	15	11	17	202
		Slow run	0.14	0.16	0.38	0.84	not significant		−1.98	−5.38	28	7	7	36
	Hind limb	Walk	0.38	0.09	<0.01	0.04	isometry	positive allometry	−2.47	−6.89	11	9	13	152
		Slow run	0.39	0.17	0.03	0.20	isometry	positive allometry	−2.69	−3.59	35	7	7	43
GSM (M^0.00^)	Forelimb	Walk	0.08	0.06	0.17	n/a	not significant		−0.38	−15.33	15	10	16	125
		Slow run	−0.04	0.11	0.72	n/a	not significant		−0.05	−9.45	30	5	6	37
	Hind limb	Walk	0.03	0.09	0.74	n/a	not significant		−0.25	−10.78	11	8	13	125
		Slow run	0.01	0.08	0.96	n/a	not significant		−0.11	−12.38	28	6	6	25
limb angle at impact (M^0.00^)	Forelimb	Walk	−0.04	0.02	0.12	n/a	not significant		1.40	−52.00	14	10	16	148
		Slow run	−0.19	0.10	0.06	n/a	not significant		1.62	−11.90	31	6	6	38
	Hind limb	Walk	−0.02	0.03	0.56	n/a	not significant		1.40	−34.00	11	9	13	145
		Slow run	−0.06	0.06	0.29	n/a	not significant		1.45	−17.67	31	6	6	38

Shown: scaling exponent (from LMM analysis), exponent standard error, p value determining if the slope of the data is significantly different from zero, p value determining if the slope of the data is significantly different from the isometric prediction, scaling analysis conclusion, scaling trend (if conclusion is not significantly different from isometry), intercept, t statistic (observed slope vs. slope predicted by isometry), degrees of freedom, number of species, number of individuals and number of impacts (i.e. instances of foot impact analysed) per impact variable. The exponent b is for the equation: log y = b log(M_b_)+log(a); where b is the slope (exponent); a is the elevation (y-intercept); and y is the impact parameter of interest. Asterisks denote an exponent that is significantly different from isometry.

### Peak Impact Force Amplitude (M_b_^1.00^)

In support of our hypothesis, our results revealed that peak vertical impact force amplitude typically scaled with isometry (Fig. 2a). Peak horizontal impact force amplitude on the other hand typically scaled lower than isometry predicts (∼M_b_^0.76±0.05^, p<0.01 forelimb walk; ∼M_b_^0.78±0.06^, p<0.01 and ∼M_b_^0.66±0.06^, p<0.01 for hind limb walk and slow run respectively; Fig. 2b, Table 1). Where we were unable to exclude isometry, consistent trends towards negative allometry were evident. In general, the forelimb impact force amplitudes were greater than the hind limb amplitudes, although these differences were not significant in the majority of species (Mann Whitney U Test, Tables S1 and S3). As expected, a gait shift from walking to slow running caused impact force amplitudes to increase; however, this increase was typically not significant (Mann Whitney U Test). The (median ± IQR) maximum vertical impact force amplitude (for both limbs) did not exceed 0.71±0.09 times body weight_,_ whereas the maximum horizontal impact force amplitude ranged from 0.04±0.01 times body weight in the largest mammals to 0.31±0.10 times body weight in the smallest mammal (Table S2 and S4).

### Impact Velocity (M_b_^0.16^)

Forefoot vertical impact velocity did not scale significantly, and although hindfoot vertical impact velocity showed allometric trends, it scaled according to isometry; our hypothesis that impact velocity would scale<M_b_^0.16^ is therefore rejected (Fig. 3a, Table 2). Although forelimb impact velocity in smaller species appeared to be higher than hindlimb impact velocity, the difference between limbs was not significant (Mann Whitney U Test, Table S5). Vertical foot impact velocities (median ± IQR) remained below 1.17±0.92 m s**^−^** ^1^ during walking speeds and 2.07±0.46 m s**^−^** ^1^ during slow running speeds (Table S6).

Horizontal foot velocity at impact did not scale significantly in either limb (Fig. 3b, Table 2). During slow running speeds, the hind limbs of all species appeared to impact the ground at faster horizontal velocities than forelimbs as we hypothesised (Table S7), however this difference was not significant in the majority of species (Mann Whitney U Test). Horizontal impact velocities (median ± IQR) remained below 2.99±0.58 m s**^−^** ^1^ during walking speeds and 4.38±2.54 m s**^−^** ^1^ during slow running speeds (Table S8). Vertical and horizontal impact velocities increased with a gait shift from walking to running, although these differences were not typically significant (Mann Whitney U Test).

### Impact Duration (M_b_^0.17^)

We hypothesized that impact duration would scale>M_b_^0.17^; whereas forefoot impact duration did not scale significantly, hind limb impact duration scaled higher than expected from isometry during walking (∼M_b_^0.38±0.09^, p<0.01), but scaled according to isometry during slow running (Fig. 4, Table 2). A gait shift from walking to running caused impact duration to decrease, but this difference was not significant (Mann-Whitney U Test, Table S9). Excluding the elephant, impact duration for all species during walking was ∼24 milliseconds (ms), whereas during slow running it was ∼18 ms (Table S10).

### M*_eff_* (M_b_^0.83^)

Although *M_eff_* showed allometric trends, this parameter scaled according to isometry as hypothesized (see Fig. 5, Table 1). The fitted models for each limb intersect, which suggests that smaller species (below ∼750 kg M_b_) have greater forelimb M*_eff_,* whereas larger species have greater hind limb M*_eff_*; however the standard errors associated with model fit (Fig. 6a and 6b) meant that these limb differences were not significant, even at extreme body sizes (Table S11). The M*_eff_* appeared to increase with a gait shift from walking to running (except for elephant hind limb M*_eff_*), although the differences were again not significant. The average *M_eff_* value for all species was ∼1.3% M_b_ (Table S12).

### Loading Rate (M_b_^0.83^)

In support of our hypothesis that loading rate would scale<M_b_^0.83^, the maximum loading rate (averaged over a window of 0.5% of stance phase throughout the impact period) scaled lower than isometry predicts (forelimb: ∼M_b_, ^0.68±0.05^, p<0.05 and ∼M_b_^0.59±0.06^, p<0.01; hind limb ∼M_b_^0.65±0.07^, p<0.05 and ∼M_b_^0.51±0.08^, p<0.01 at walking and slow running respectively, Fig. 7a, Table 1). In both gaits, the forelimb rate of force application was consistently greater than the hind limb rate of force application. This difference was significant in the majority of species at walking speeds but only in one out of seven (1/7) species at slow running speeds (Mann Whitney U Test, Tables S13 and S14).

Similarly, excluding the forelimb during slow running speeds, the maximum instantaneous loading rate scaled lower than isometry predicts as hypothesized (forelimb walk ∼M_b_^0.64±0.08^, p<0.05; hind limb ∼M_b_^0.56±0.11^, p<0.05 and ∼M_b_^0.49±0.12^, p<0.01 at walking and slow running respectively, Fig. 7b, Table 1). Although limb differences were not significant, a shift in gait from walking to running caused the maximum instantaneous rate of force application to increase; this difference was significant in five out of eight (5/8) and three out of seven (3/7) species in the fore- and hind limb respectively (Table S15). The sheep exhibited the highest loading rate (in excess of 100 times M_b_ s**^−^** ^1^), while the dromedary camel exhibited the lowest loading rate (below 3 times M_b_ s**^−^** ^1^, Table S16).

### Peak Vertical GRF Amplitude (M_b_^1.00^)

Excluding the forelimb during walking speeds, in support of our hypothesis, the peak vertical GRF amplitude scaled lower than isometry predicts (forelimb slow run ∼M_b_^0.72±0.10^, p<0.05; and hind limb ∼M_b_^0.85±0.06^, p<0.05 and ∼M_b_^0.76±0.08^, p<0.01 at walking and slow running respectively, Fig. 8, Table 1). Forelimb GRF amplitudes were consistently greater than hind limb amplitudes; this difference was significant in six out of nine (6/9) and three out of six (3/6) species at walking and slow running speeds respectively (Mann Whitney U Test, Table S17). As expected, the peak vertical GRF increased with speed, with the greatest (median ± IQR) amplitude reaching 2.87±0.63 times body weight during slow running speeds in the blackbuck antelope (Table S18).

### Impact Impulse (M_b_^1.17^)

The vertical impact impulse (Fig. 1d) generated by both the fore- and hind limbs ranged from ∼1–4% M_b_ s**^−^** ^1^ and scaled according to isometry as hypothesized (Fig. 9a, Table 1). Interestingly, whereas the forelimb impact impulses showed a trend for negative allometry (M_b_^0.99±0.11^, p = 0.13; M_b_^1.05±0.22^, p = 0.59, walk and slow run respectively), the hind limb impact impulses showed a trend for positive allometry (M_b_^1.38±0.14^, p = 0.15; M_b_^1.21±0.22^, p = 0.86 walk and slow run respectively). During slow running, the forelimb vertical impact impulse was consistently greater than the hind limb impact impulse, although these differences were typically not significant (Mann Whitney U Test, Tables S19 and S20).

The (absolute) horizontal forelimb impact impulse (Fig. 1e) scaled lower than isometry predicts during walking speeds (∼M_b_^0.86±0.12^, p<0.05), but scaled according to isometry during running speeds and at both speeds in the hind limb (Fig. 9b, Table 1). During walking speeds, although impact impulses appeared greater in the forelimbs of species below ∼750 kg M_b_ these limb differences were not significant (Fig. 10a and 10b, Tables S21 and S22).

### Total Decelerative/Accelerative Impulse (M_b_^1.17^)

The total decelerative impulse for the entire stance phase (Fig. 1f) scaled lower than isometry predicts in the forelimb (∼M_b_^0.90±0.09^, p<0.05; ∼M_b_^0.83±0.13^, p<0.05, walk and slow run respectively, Fig. 11a); median decelerative impulses were ∼8–9% M_b_ in the blackbuck antelope, whereas they were ∼1–2% M_b_ in the elephant (Table S24). Although isometry could not be excluded in the hind limb decelerative impulses, we observed a concurrent trend for negative allometry (Table 1). Forelimb decelerative impulses were consistently greater than hindlimb decelerative impulses, these differences were significant in five out of nine (5/9) species during walking and two out of nine (2/9) species during slow running (Mann Whitney U Test, Table S23).

Excluding the forelimb during walking speeds, the total accelerative impulse (Fig. 1g) scaled lower than isometry predicts (forelimb ∼M_b_^0.90±0.13^, p<0.05; hindlimb ∼M_b_^0.81±0.11^, p<0.05; ∼M_b_^0.77±0.14^, p<0.05, walk and slow run respectively, Fig. 11b). Median accelerative impulses were ∼7–8% M_b_ in the smallest hoofed mammal and ∼1% M_b_ in the largest hoofed mammal (Table S26). Isometry could not be excluded in the forelimb during walking speeds, although this limb showed a trend for slight negative allometry (Table 1). During walking, the fitted models appear to intersect ∼300 kg M_b_ (Fig. 12)_,_ however when the large standard errors are considered limb differences are less obvious (Table S25).

### GSM (M_b_^0.00^)

GSM did not scale significantly in either limb at either gait (Table 2, Table S27). Notably however, median GSM values were consistently high in the elephant (92–98%), indicating an almost smooth landing, whereas values obtained from the deer during walking speeds were markedly low (13% and 15%).

### Limb Impact Angle (M_b_^0.00^)

Limb angle at impact did not scale significantly in either limb at either gait (Table 2). Forelimb impact angle for all species (median ± IQR) was 21±7° and 17±6° whereas hind limb impact angle was 24±4° and 22±4° (walk and slow run respectively).

## Discussion

Our study compared how features of foot impact change with body size in hoofed mammals, for whom foot health is a major global welfare concern [22]–[27]. In support of our primary hypothesis, we found that peak impact force amplitude scales according to isometry (or lower than isometry predicts).We expected force amplitudes to be moderated through allometric scaling patterns in related impact parameters, which would fit the general theory that in order to maintain tissue safety factors, larger animals exhibit size-dependent changes [6]–[11], [19]. Significant allometry was found in a limited number of parameters. However, typically, statistically significant allometry was found in only one limb (or at one speed), with consistent, albeit non-significant, allometric trends evident in the remaining limb/speed.

### Isometry vs. Allometry

In general, (as hypothesized) peak vertical impact force amplitudes scale according to isometry while peak horizontal force amplitudes scale lower than isometry predicts. This means that larger hoofed mammals experience relatively similar (or lower) peak impact forces relative to what smaller hoofed mammals experience. Considering these impact forces are applied less often [8], [9], it seems that the increased mass associated with large body-size does not simply translate to increased loading during foot-ground contact. The peak impact forces obtained by this study are lower than those previously reported in humans [1], [4], and when compared to the maximal vertical force amplitudes generated around mid-stance, impact forces are remarkably low.

While we use the Froude number to roughly gauge dynamic similarity, allowing comparison of smaller and larger species at equivalent speeds, it is certain that dynamic similarity does not hold perfectly true across the full size range studied here [28], [34]. Equally, geometric similarity of foot impact parameters (especially foot geometry and material properties) may not be tenable, particularly for very large species and those that deviate from a truly hoof like morphology and unguligrade foot posture. Considering these potential irregularities, attempting to fit a single model to the data may seem ineffective, however we feel that our identifications of common patterns in the evolution of size and locomotion are nonetheless valuable.

*M_eff_* typically scales according to isometry, although we observe a trend for positive allometry in the hind limb. This suggests that the amount of foot-limb mass that contributes to generating impact force remains relatively similar (or increases) with increasing body mass. The *M_eff_* values we obtained from our set of mammalian quadrupeds were expected to be lower than those previously reported for bipedal humans, because quadrupeds have reduced distal limb mass and share the load between multiple limbs. However, in terms of percentage body mass, *M_eff_* values for single foot impacts generated here (<5% M_b_) are very similar to the ∼5% M_b_ values found in (single) human foot impacts [29], [30]. Interestingly, the scaling of *M_eff_* is not consistently reflected in the scaling of impact force amplitude, which implies that foot acceleration is altered (see equation (1)). This study did not quantify the rate of velocity change, but simply the velocity change caused by foot impact.

Unlike the relatively fixed mass of a real limb segment, *M_eff_* describes the amount of limb mass that generates impact force. The impulse momentum method derives a mean *M_eff_* value for the impact period, whereas in reality this parameter is likely to vary dynamically depending on neuromuscular control. This oversimplification makes interpreting the biological relevance of *M_eff_* values difficult to ascertain when viewed in isolation, hence our combined approach that investigates many of the parameters involved in foot impact dynamics. It is nonetheless a necessary oversimplification due to the complications imposed by calculating instantaneous values for different species, footfall timings and capture frequencies.

We expected foot velocity just prior to impact to scale with negative allometry; however, this parameter scales according to isometry and even trends towards positive allometry. This trend suggests that the hind feet of larger hoofed mammals impact the ground at equivalent (or higher) vertical velocities than the hind feet of smaller hoofed mammals. It remains unclear how forelimb impact velocities change as a result of size, but considering that limb lengths remain similar (in geometrically similar animals) and limb angles may scale ∼M_b_ **^−^** ^0.10^ as [19] reported, pendulum law infers that horizontal impact velocities scale with negative allometry.

Ground speed matching (GSM) did not scale significantly. This value would have described where each species falls between two idealized extremes– one being a foot landing smoothly without any impact and the other being a foot moving (sliding) with the same velocity as the centre of mass [31] - in the Results we noted how elephants seem to come closest to matching the smooth landing extreme. Our allometric findings for impact force amplitude favour the inference that GSM values should get closer to 1 (smooth landing with no impact) with increasing body size, as displayed by elephants in our sample.

We expected impact duration to scale allometrically; we found that this parameter scales according to isometry, but with a trend for positive allometry (in the hind limb). This hints that the period over which impact loading occurs is similar (or longer) in the hind limbs of larger hoofed mammals, which stands to reason, because stance duration generally increases with body size [8], [9]. While we assumed the hoofed mammals included in this study have a similar (mainly artiodactyl) foot design, the increased volume of viscoelastic tissue (obvious in *Elephas*, *Camelus* and *Vicugna* feet) is likely to alter the behaviour of the tissues under load, as well as increase foot compliance and extend impact duration [1], [28], [32]. Additionally, on a mass-specific basis, some larger animals may use more compliant limbs as faster speeds [33], which would prolong impact duration. We were unable to quantify limb stiffness or motion during foot impact, as that calculation would require 3D motion data and would involve difficulties defining limb stiffnesses for fore- and hind limbs in multiple gaits.

In agreement with [35], both the maximum average loading rate (calculated over a 0.5% window during impact loading) and the maximum instantaneous loading rate (typically) scale lower than isometry predicts. From this finding, we infer that smaller hoofed mammals undergo higher foot tissue strain rates during impact loading. Rate of loading has importance when considering the mechanical behaviour of musculoskeletal tissues during impact – faster loading rates are typically associated with increased tissue stiffness [36], which could enhance impact force transmission.

Although sensorimotor responsiveness is not maintained with size [13] and thus larger animals cannot respond to external stimuli as fast as smaller animals, we found that the time to impact peak occurred up to 90 ms after initial foot contact. This period exceeds the latency period of muscle and therefore suggests some (smaller) hoofed mammals may feasibly be able to actively alter joint flexion, limb stiffness or utilize active damping within the impact period in order to attenuate impact. Further work is necessary in order to determine the involvement of these active control mechanisms in extending impact duration.

Because impulse is the integral of force with respect to time, the isometry exhibited in the vertical impact impulses are somewhat expected. Interestingly though, whereas the forelimb impact impulses trend towards negative allometry, the hind limb impact impulses trend towards positive allometry. These trends suggest that during the impact phase, larger hoofed mammals experience a smaller change of momentum in the forelimb but a larger change in momentum in the hind limb than smaller hoofed mammals do. In contrast, the horizontal impulses during impact (and indeed throughout the entire stance phase) typically scale lower than expected from isometry. This pattern suggests that larger hoofed mammals experience a relatively smaller change in horizontal momentum during impact loading than smaller hoofed mammals do. Furthermore, larger hoofed mammals may experience a relatively smaller change in horizontal momentum when decelerating/accelerating than smaller species do.

We expected limb impact angle to scale lower than isometry predicts, in line with previous work [19]; however, we did not observe a significant scaling relationship, suggesting that impact angle (in these species, at walking and slow running speeds) is invariant to increasing body size. Indeed, it should be acknowledged that the former study did not report its confidence intervals for scaling exponents and thus may not have found truly significant allometry of limb angles at limb contact.

### Forelimb vs. Hind Limb Impacts

Congruent with previous work and in line with quadrupedal mass distribution, our study suggests that, in hoofed mammals, the forelimbs experience relatively greater vertical impact (and mid-stance) force amplitudes than the hind limbs [18], [28]. Related features of foot impact help to explain why this might be so; if the forefoot has greater *M_eff_* and travels at a faster vertical velocity just prior to impact (as our data for smaller hoofed mammals suggest), greater impact forces are to be expected in the forelimb. Additionally, if forelimb impact durations are shorter, it follows that the rate of force application must increase. In larger hoofed mammals, it seems that increased impact duration counters greater *M_eff_* and impact velocity in the hind limb (in line with [37]). The overall result is that force amplitudes in the forelimb remain higher than those in the hind limb.

Many impact parameters were highly variable even within species, rendering it difficult to conclusively exclude isometry from much of the analysis. Similarly, the standard errors associated with the fitted models generate substantial overlap between conditions analysed. Regardless, we have revealed some of the mechanical strategies that larger animals employ to counter the increased mass associated with large size and have additionally illuminated striking differences between how features of foot impact scale in the forelimb versus how they scale in the hind limb, which certainly relates to differential limb function in quadrupedal mammals. The forelimb’s greater capacity for braking and weight support is reflected by this limb experiencing greater peak vertical impact (and mid-stance) force amplitudes. In contrast, the hind limb’s propensity for propulsion is reflected in the positive allometric trends consistently exhibited in vertical impact impulse, *M_eff_* and impact velocity.

### Conclusion

Considering previously described size-related changes in limb posture, duty factor [6] (and in very large animals) bone robusticity [7], the (generally) isometric scaling patterns revealed by our study support the conclusion that increased body size in hoofed mammals is unlikely to increase risk of fatigue accumulation induced by foot impact. Although the relative magnitudes of impact forces are similar regardless of body size, the frequency at which these impact loads are applied is reduced with size [8]. We speculate that extending impact duration is imperative to moderating impact force amplitudes in larger animals, since there is evidence of positive allometric trends in hind limb foot impact parameters.

This study suggests that limb-dependent scaling patterns exist; for each parameter the forelimb typically exhibits a higher intercept and a lower scaling exponent than the hind limb, causing the slopes to intersect. The standard errors associated with the fitted models and this intersection prevent the differences between fore- and hind limb scaling from being statistically significant, but our findings hint that body size may alter limb dominance. The magnitude of forelimb parameters appears greater for very small hoofed mammals, whereas the magnitude of hind limb impact parameters appears greater for very large hoofed mammals. We speculate that these disparities indicate size-dependent alterations in mechanical limb function at extreme body sizes. Finally, increasing body mass appears to have less effect on forelimb impact mechanics; this may signify changes in mass-distribution, or perhaps that forelimb parameters are more tightly controlled. Our study, by elucidating these patterns for the first time, highlights the neglected importance of a comparative approach in understanding the principles that relate impact dynamics to limb morphology, foot design and locomotor behaviour.

### Methods

Data from three experimental setups were combined for this study. Lab-based data from four species (*Ovis aries* (sheep), *Sus scrofa domestica* (pig), *Vicugna pacos* (alpaca), *Equus caballus* (horse)) were acquired using Qualisys Oqus cameras (250 Hz or 167 Hz; Qualisys AB) and Kistler force platforms (1000 Hz or 500 Hz; Kistler Instruments Ltd). Field-based data from six species (*Antilope cervicapra* (blackbuck antelope), *Addax nasomaculatus* (addax), *Cervus elaphus* (red deer), *Bos taurus* (bull), *Camelus dromedarius* (dromedary camel), *Giraffa camelopardalis* (giraffe)) were acquired using AOS High Speed Cameras (250 Hz; AOS Technologies AG) and AMTI force platforms (200 Hz; Advanced Mechanical Technology Inc.). *Elephas maximus* (Asian elephant) data were obtained by Ren *et al*. [28] in Thailand. Ethical approval for all experiments was granted by the Royal Veterinary College’s Ethics and Welfare Committee (permit number: URN 2012 1146-2009).

All subjects were selected on the basis of age, body mass and musculoskeletal health and were adults. Data collection continued until at least 10 apparently steady-state trials (minimal net acceleration/deceleration) with single foot-plate contacts were observed at both walking and slow running speeds. A list of mammals used in this study is provided in Table 3.

**Table 3:** Subject information.

		subject	hip height (m)	body mass (kg)
*Elephas maximus*	Asian elephant	1	1.77	3157
*Elephas maximus*	Asian elephant	2	1.51	2318
*Elephas maximus*	Asian elephant	3	1.40	1984
*Giraffa camelopardalis*	Giraffe	1	2.20	1058
*Camelus dromedarius*	Dromedary camel	1	1.44	992
*Bos Taurus*	Bull	1	1.23	955
*Equus caballus*	Horse	1	1.11	562
*Equus caballus*	Horse	2	0.96	318
*Cervus elaphus*	Red deer	1	0.90	134
*Vicugna pacos*	Alpaca	1	0.69	72
*Vicugna pacos*	Alpaca	2	0.71	67
*Addax nasomaculatus*	Addax	1	0.82	53
*Sus domestica*	Pig	1	0.46	52
*Sus domestica*	Pig	2	0.40	48
*Ovis aries*	Sheep	1	0.58	35
*Ovis aries*	Sheep	2	0.60	33
*Antilope cervicapra*	Blackbuck antelope	1	0.63	18

Reflective markers were placed on the hip/shoulder and lateral hoof for tracking limb and foot motions. Marker trajectories were either tracked using Qualisys Track Manager (QTM) or a custom Matlab (Mathworks Inc.; Natick, MA, USA) digitizing tool [38] in order to convert the acquired 2D data to 3D coordinates. Raw force data were zeroed by subtracting the mean noise from each channel at a period when there was nothing on the force platform. Raw force data were summed to get Fx, Fy, Fz and a threshold value of 5N was applied to cut the data into single stances (foot on: foot off). Force data were low-pass filtered (Kistler; 100 Hz single pole RC filer, AMTI; 200 Hz 2 pole filter). Foot timings from kinematic data were matched to stance timings in kinetic data in order to identify and label individual limb contacts.

Single foot contact data were selected for further processing. In some instances, incomplete stance phases (e.g., missing some late stance phase data due to second foot contacts with the plate) were used to increase the data available for analysis; to do this, stance duration was determined using foot vertical displacement data. Kinetic and kinematic data were subsequently analysed using custom written Matlab code.

We calculated M*_eff_* using the impulse momentum method [12], [29], as follows (equation 1):
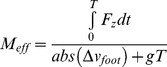
(1)Where 0 is the beginning of the impact impulse –i.e., the instant of initial foot contact (where the vertical force exceeded 5N) is the end of the impact impulse –i.e. the duration of the impact (where the vertical impact force peaked) and, 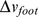 is the absolute change in vertical velocity of the foot.

In addition, we calculated peak vertical impact force, peak vertical ground reaction force (GRF), vertical (and horizontal) foot velocity just prior to foot impact, vertical (and horizontal) impact impulse, impact duration and rate of loading. Maximum instantaneous loading rate was calculated using the biggest frame to frame increase in force during the impact period. In addition, we calculated maximum loading rate using a rolling window of 0.5% stance throughout the impact period. We also calculated limb angle at foot impact using basic trigonometry [19], and we calculated ground speed matching (GSM); i.e., the ratio between the resultant centre of mass velocity and resultant foot velocity [31]. Because many of our data were not normally distributed, we report median ± interquartile ranges (IQR).

**Figure 13:**
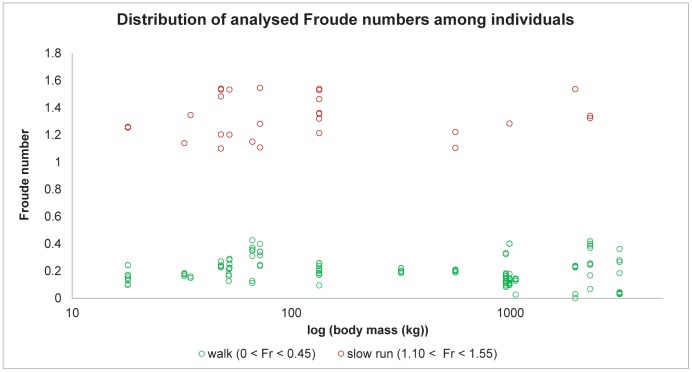
Distribution of analysed Froude numbers among individuals. Green unfilled markers denote walk data (fore- and hind limbs), red unfilled markers denote slow run data (fore- and hind limbs).

For comparison among and within taxa of different sizes [39], the Froude number, a roughly dynamically similar speed, was calculated using Froude number (*u* ^2^/*gL* where *u* is velocity, *g* is the acceleration due to gravity and *L* is hip height). Froude bins were defined as Fr 0.01–0.45 (walking) and Fr 1.10–1.55 (slow running). Figure 13 shows the distribution of analysed Froude numbers among species and indicates that the data samples were roughly comparable, which our statistical analyses took into account (see below). Specific footfall patterns were not quantified here, but qualitatively all species were using a lateral sequence walk at walking speeds and most used a trot at running speeds (exceptions being elephants and antelope “ambling” and camel/alpacas pacing).

This study makes several assumptions: that hoofed mammals move with dynamically similar locomotion at equivalent Froude numbers ([39]; as above); that the hoofed mammals we include have a similar ungulate foot structure (elongated metapodials with one or more subvertically-oriented digits terminating in relatively small, rigid hooves, claws or nails); and that material properties [10], [37] and foot contact area [40] scale isometrically (the latter two assumptions being supported to some degree by prior studies). We also include some semi-unguligrade species in our analysis (*Vicugna pacos* (alpaca), *Camelus dromedarius* (camel), and *Elephas maximus* (elephant)).

Statistical analyses were conducted in *R* programming software (R Foundation for Statistical Computing). Linear mixed-effect models (LMM) were fitted for each impact variable, using body mass as the fixed effect and individual (or species) as the random effect. Separate models were fitted for each limb (fore- and hind limb) and for each gait (walk and slow run), see below for (forelimb walk) model details:

To test the fitted model against a slope of zero:
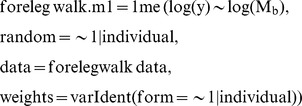



To test the fitted model against the predicted isometric slope (in this case M_b_^0.83^):
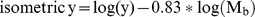


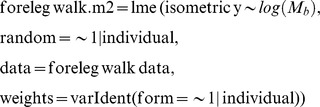
where y is the impact variable of interest.

The residual terms were normalised using a species-dependant or individual-dependant term. Model fit was verified using QQ plots and fitted residual plots and statistical significance was set at p<0.05.To test the effect of speed and limb we used a Mann-Whitney U Test, the significance level was set at 0.05, and was adjusted using a post hoc Bonferroni correction to 0.02 to avoid Type I errors. The total number of foot impacts analysed ranged from 3 to 38 per species depending on impact parameter (see Table 1; kinetic impact dynamic parameters and Table 2; kinematic impact parameters for further details). An additional study of phylogenetically independent contrasts was conducted to check for evolutionary non-independence of species data points but did not uncover results that substantially differ from those reported here (see Methods S1, Table S28 and Figures S1–S3).

## Supporting Information

Figure S1 **Phylogenetic tree used for independent contrasts analysis with branch lengths set to 1 unit each.**
(DOCX)

Figure S2 **Results of independent contrasts analysis using branch lengths equal to 1 unit.**
(DOCX)

Figure S3 **Results of independent contrasts analysis: branch lengths set using Pagel’s transform.**
(DOCX)

Table S1 **Peak vertical impact force amplitude– MannWhitney U Test outcomes comparing limb and speed effects.**
(DOCX)

Table S2 **Peak vertical impact force amplitude: values are expressed as multiples of body weight (x BW); median amplitude (IQR) per species is shown.**
(DOCX)

Table S3 **Peak horizontal impact force amplitude– MannWhitney U Test outcomes comparing limb and speed effects.**
(DOCX)

Table S4 **Peak horizontal impact force amplitude: values are expressed as multiples of body weight (x BW); median amplitude (IQR) per species is shown.**
(DOCX)

Table S5 **Vertical impact velocity– MannWhitney U Test outcomes comparing limb and speed effects.**
(DOCX)

Table S6 **Vertical impact velocity: values are expressed in metres per second; median (IQR) per species is shown.**
(DOCX)

Table S7 **Horizontal impact velocity– MannWhitney U Test outcomes comparing limb and speed effects.**
(DOCX)

Table S8 **Horizontal impact velocity: values are expressed in metres per second; median (IQR) per species is shown.**
(DOCX)

Table S9 **Impact duration– MannWhitney U Test outcomes comparing limb and speed effects.**
(DOCX)

Table S10 **Impact duration: values are expressed in milliseconds; median (IQR) per species is shown.**
(DOCX)

Table S11 **M** ***_eff_*** **– MannWhitney U Test outcomes comparing limb and speed effects.**
(DOCX)

Table S12 **M** ***_eff_*** **values are expressed as a percentage of body weight; median (IQR) per species is shown.**
(DOCX)

Table S13 **Maximum average loading rate (calculated over a window of 0.5% stance during the initial 25% stance)– MannWhitney U Test outcomes comparing limb and speed effects.**
(DOCX)

Table S14 **Maximum average loading rate (calculated over a window of 0.5% stance during the initial 25% stance): values are expressed in body weights per seconds (M_b_ s^−1^); median loading rate (IQR) per species is shown.**
(DOCX)

Table S15 **Maximum instantaneous loading rate– Mann Whitney U Test outcomes comparing limb and speed effects.**
(DOCX)

Table S16 **Maximum instantaneous loading rate: values are expressed in body weights per second (M_b_ s^−1^); median loading rate (IQR) per species is shown.**
(DOCX)

Table S17 **Peak vertical ground reaction force (GRF) amplitude– MannWhitney U Test outcomes comparing limb and speed effects.**
(DOCX)

Table S18 **Peak vertical ground reaction force (GRF) amplitude: values are expressed as multiples of body weight; median amplitude (IQR) per species is shown.**
(DOCX)

Table S19 **Vertical impact impulse– MannWhitney U Test outcomes comparing limb and speed effects.**
(DOCX)

Table S20 **Vertical impact impulse: values are expressed as percentage of body weight per second (%BW s): median impact impulse (IQR) per species is shown.**
(DOCX)

Table S21 **(abs) horizontal impact impulse– MannWhitney U Test outcomes comparing limb and speed effects.**
(DOCX)

Table S22 **(abs) horizontal impact impulse: values are expressed in percentage bodyweight per second (%BW s); median impact impulse (IQR) per species is shown.**
(DOCX)

Table S23 **Total decelerative impulse– MannWhitney U Test outcomes comparing limb and speed effects.**
(DOCX)

Table S24 **Total decelerative impulse– values are expressed in percentage bodyweight per second (%BW s); median impact impulse (IQR) per species is shown.**
(DOCX)

Table S25 **Total accelerative impulse– MannWhitney U Test outcomes comparing limb and speed effects.**
(DOCX)

Table S26 **Total accelerative impulse– values are expressed in percentage bodyweight per second (%BW s); median impact impulse (IQR) per species is shown.**
(DOCX)

Table S27 **Ground speed matching (GSM): values represent ratio between the resultant centre of mass velocity and resultant foot velocity; median (IQR) per species is shown.**
(DOCX)

Table S28 **Results of phylogenetically independent contrasts analysis.**
(DOCX)

Methods S1 **Phylogenetically independent contrasts analysis.**
(DOCX)

References S1 (DOCX)
