# Supplementary material for: Size-Related Changes in Foot Impact Mechanics in Hoofed Mammals
Source: PLoS One. 2013 Jan 30;8(1):e54784. doi: 10.1371/journal.pone.0054784 (PMC3559824; doi:10.1371/journal.pone.0054784)
Supplement: Figure S1 — Phylogenetic tree used for independent contrasts analysis with branch lengths set to 1 unit each. (DOCX) [file pone.0054784.s001.docx]

Supplementary Figure S30: Phylogenetic tree used for independent contrasts analysis with branch lengths set to 1 unit each (scale on right side). See supplementary text S28 for details.
