# Supplementary material for: Size-Related Changes in Foot Impact Mechanics in Hoofed Mammals
Source: PLoS One. 2013 Jan 30;8(1):e54784. doi: 10.1371/journal.pone.0054784 (PMC3559824; doi:10.1371/journal.pone.0054784)
Supplement: Figure S2 — Results of independent contrasts analysis using branch lengths equal to 1 unit. (DOCX) [file pone.0054784.s002.docx]

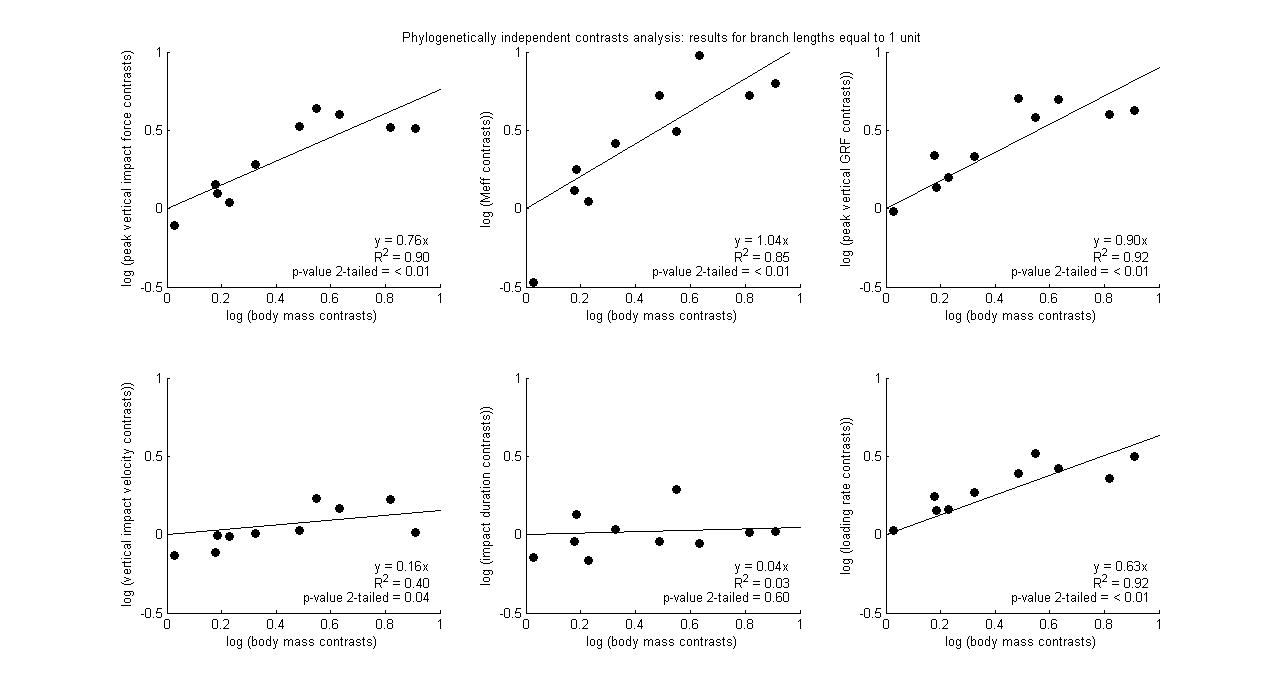


Supplementary Figure S31: Results of independent contrasts analysis: using branch lengths equal to 1 unit. These results focus on logarithmically transformed data from the forelimb at walking speeds; peak vertical impact force, M*_eff_*, peak vertical ground reaction force, vertical impact velocity, impact duration and loading rate (calculated over a 0.5% window during the impact period). This figure, when considered alongside Figure S30 and Table S29 shows that even if there is a phylogenetic bias in our data, correcting for it does not alter our conclusions appreciably and is statistically a worse fit to the data than non-phylogenetically adjusted data, which the main text focuses on.
