# Supplementary material for: Size-Related Changes in Foot Impact Mechanics in Hoofed Mammals
Source: PLoS One. 2013 Jan 30;8(1):e54784. doi: 10.1371/journal.pone.0054784 (PMC3559824; doi:10.1371/journal.pone.0054784)
Supplement: Figure S3 — Results of independent contrasts analysis: branch lengths set using Pagel’s transform. (DOCX) [file pone.0054784.s003.docx]

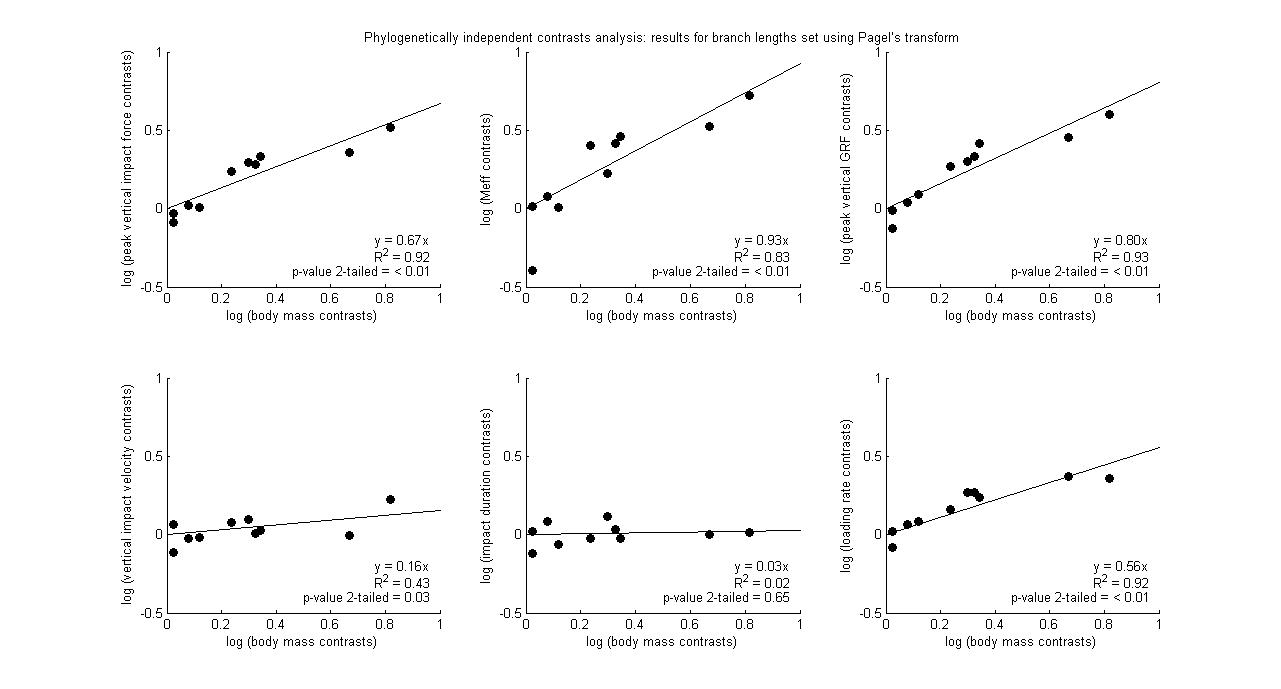


Supplementary Figure S32: Results of independent contrasts analysis: branch lengths set using Pagel’s transform. These results focus on logarithmically transformed data from the forelimb at walking speeds; peak vertical impact force, M*_eff_*, peak vertical ground reaction force, vertical impact velocity, impact duration and loading rate (calculated over a 0.5% window during the impact period). See also Figure S31 caption.
