# Supplementary material for: Size-Related Changes in Foot Impact Mechanics in Hoofed Mammals
Source: PLoS One. 2013 Jan 30;8(1):e54784. doi: 10.1371/journal.pone.0054784 (PMC3559824; doi:10.1371/journal.pone.0054784)
Supplement: Table S1 — Peak vertical impact force amplitude– MannWhitney U Test outcomes comparing limb and speed effects. (DOCX) [file pone.0054784.s004.docx]

Supplementary Table S1: peak vertical impact force amplitude-- MannWhitney U Test outcomes comparing limb and speed effects. * denotes significant differences between fore- and hind limbs, or between walk and slow run.

|  |  |  |  |  |  |
| --- | --- | --- | --- | --- | --- |
|  |  | **p value** | **Total N** | **Mann-Whitney U** | **Z** |
|  |  |  |  |  |  |
| Forelimb walk versus Hindlimb walk | Sheep | 0.057 | 25 | 43.0 | -1.904 |
|  | Pig | 0.040 | 35 | 90.0 | -2.053 |
|  | Addax | 0.728 | 15 | 25.0 | -0.347 |
|  | Alpaca | 0.519 | 25 | 40.5 | -0.646 |
|  | Deer | 0.001* | 48 | 122.0 | -3.416 |
|  | Horse | 0.001* | 56 | 188.0 | -3.343 |
|  | Bull | 0.534 | 44 | 215.0 | -0.623 |
|  | Dromedary | 0.242 | 32 | 93.0 | -1.170 |
|  | Elephant | <0.001* | 48 | 84.0 | -4.206 |
| Forelimb run versus Hindlimb run | Sheep | 0.121 | 9 | 3.0 | -1.549 |
|  | Pig | 0.923 | 17 | 35.0 | -0.096 |
|  | Alpaca | 0.096 | 8 | 1.0 | -1.667 |
|  | Deer | 0.105 | 20 | 27.0 | -1.620 |
|  | Horse | 0.011* | 14 | 3.5 | -2.536 |
|  | Elephant | 0.513 | 6 | 3.0 | -0.655 |
| Forelimb run versus Forelimb walk | Antelope | 0.315 | 24 | 20.0 | -1.004 |
|  | Sheep | 0.014* | 15 | 1.0 | -2.454 |
|  | Pig | 0.007* | 24 | 20.0 | -2.694 |
|  | Alpaca | 0.051 | 26 | 28.0 | -1.947 |
|  | Deer | 0.012* | 33 | 40.0 | -2.521 |
|  | Horse | 0.002* | 33 | 7.0 | -3.163 |
|  | Elephant | 0.005* | 27 | 0.0 | -2.777 |
| Hindlimb run versus Hindlimb walk | Sheep | 0.007* | 19 | 8.0 | -2.719 |
|  | Pig | 0.001* | 28 | 0.0 | -4.206 |
|  | Alpaca | 0.699 | 7 | 4.0 | -0.687 |
|  | Deer | 0.001* | 35 | 35.0 | -3.579 |
|  | Horse | 0.671 | 37 | 114.0 | -0.425 |
|  | Dromedary | 0.308 | 15 | 7.0 | -1.019 |
|  | Elephant | 0.165 | 27 | 18.0 | -1.389 |
