# Supplementary material for: Size-Related Changes in Foot Impact Mechanics in Hoofed Mammals
Source: PLoS One. 2013 Jan 30;8(1):e54784. doi: 10.1371/journal.pone.0054784 (PMC3559824; doi:10.1371/journal.pone.0054784)
Supplement: Table S2 — Peak vertical impact force amplitude: values are expressed as multiples of body weight (x BW); median amplitude (IQR) per species is shown. (DOCX) [file pone.0054784.s005.docx]

Supplementary Table S2: peak vertical impact force amplitude: values are expressed as multiples of body weight (x BW); median amplitude (IQR) per species is shown.

|  | **Forelimb Walk**  **vertical impact amplitude (x BW)** | | **Forelimb Slow Run**  **vertical impact amplitude (x BW)** | | **Hindlimb Walk**  **vertical impact amplitude (x BW)** | | **Hindlimb Slow Run**  **vertical impact amplitude (x BW)** | |
| --- | --- | --- | --- | --- | --- | --- | --- | --- |
| Antelope | 0.43 | (0.59) | 0.71 | (0.09) |  |  |  |  |
| Sheep | 0.18 | (0.09) | 0.57 | (0.15) | 0.07 | (0.07) | 0.22 | (0.21) |
| Pig | 0.18 | (0.04) | 0.25 | (0.07) | 0.16 | (0.03) | 0.25 | (0.04) |
| Addax | 0.43 | (0.15) |  |  | 0.42 | (0.33) |  |  |
| Alpaca | 0.21 | (0.08) | 0.26 | (0.24) | 0.18 | (0.01) | 0.13 | (0.10) |
| Deer | 0.14 | (0.08) | 0.28 | (0.20) | 0.10 | (0.02) | 0.16 | (0.07) |
| Horse | 0.06 | (0.05) | 0.19 | (0.09) | 0.13 | (0.05) | 0.11 | (0.01) |
| Bull | 0.07 | (0.04) |  |  | 0.06 | (0.03) |  |  |
| Dromedary | 0.05 | (0.02) |  |  | 0.05 | (0.02) | 0.05 | (0.01) |
| Giraffe | 0.16 | (0.06) |  |  |  |  |  |  |
| Elephant | 0.14 | (0.10) | 0.35 | (0.05) | 0.23 | (0.11) | 0.49 | (0.16) |
