# Supplementary material for: Size-Related Changes in Foot Impact Mechanics in Hoofed Mammals
Source: PLoS One. 2013 Jan 30;8(1):e54784. doi: 10.1371/journal.pone.0054784 (PMC3559824; doi:10.1371/journal.pone.0054784)
Supplement: Table S3 — Peak horizontal impact force amplitude– MannWhitney U Test outcomes comparing limb and speed effects. (DOCX) [file pone.0054784.s006.docx]

Supplementary Table S3: peak horizontal impact force amplitude-- MannWhitney U Test outcomes comparing limb and speed effects. * denotes significant differences between fore- and hind limbs, or between walk and slow run.

|  |  |  |  |  |  |
| --- | --- | --- | --- | --- | --- |
|  |  | **p value** | **Total N** | **Mann-Whitney U** | **Z** |
|  |  |  |  |  |  |
| Forelimb walk versus Hindlimb walk | Sheep | 0.744 | 25 | 72.0 | -0.326 |
|  | Pig | 0.947 | 35 | 150.0 | -0.066 |
|  | Addax | 0.005* | 17 | 7.0 | -2.791 |
|  | Alpaca | 0.930 | 27 | 61.5 | -0.088 |
|  | Deer | <0.001* | 47 | 65.0 | -4.477 |
|  | Horse | 0.961 | 56 | 389.0 | -0.049 |
|  | Bull | 0.474 | 44 | 211.0 | -0.717 |
|  | Dromedary | 0.002* | 32 | 41.0 | -3.165 |
|  | Giraffe | 0.127 | 8 | 0.0 | -1.528 |
|  | Elephant | 0.007* | 43 | 120.0 | -2.678 |
| Forelimb run versus Hindlimb run | Sheep | 0.439 | 9 | 0.0 | -0.775 |
|  | Pig | 0.083 | 17 | 18.0 | -1.732 |
|  | Alpaca | 0.182 | 8 | 2.0 | -1.333 |
|  | Deer | 0.247 | 20 | 33.0 | -1.157 |
|  | Horse | 0.016* | 14 | 4.5 | -2.403 |
|  | Dromedary | 0.221 | 3 | 0.0 | -1.225 |
|  | Elephant | 0.050 | 6 | 0.0 | -1.964 |
| Forelimb run versus Forelimb walk | Antelope | 0.694 | 24 | 27.0 | -0.393 |
|  | Sheep | 0.061 | 15 | 5.0 | -1.876 |
|  | Pig | 0.098 | 24 | 37.0 | -1.653 |
|  | Alpaca | 0.054 | 27 | 30.0 | -1.931 |
|  | Deer | 0.015* | 33 | 42.0 | -2.436 |
|  | Horse | 0.001* | 33 | 1.0 | -3.464 |
|  | Dromedary | 0.099 | 20 | 0.0 | -1.648 |
|  | Elephant | 0.006* | 26 | 0.0 | -2.769 |
| Hindlimb run versus Hindlimb walk | Sheep | 0.009* | 19 | 9.0 | -2.631 |
|  | Pig | 0.863 | 28 | 82.0 | -0.172 |
|  | Alpaca | 0.505 | 8 | 4.0 | -0.667 |
|  | Deer | <0.001* | 34 | 7.0 | -4.505 |
|  | Horse | 0.026 | 37 | 63.0 | -2.230 |
|  | Dromedary | 0.027 | 15 | 0.0 | -2.208 |
|  | Elephant | 0.144 | 23 | 14.0 | -1.461 |
