# Supplementary material for: Size-Related Changes in Foot Impact Mechanics in Hoofed Mammals
Source: PLoS One. 2013 Jan 30;8(1):e54784. doi: 10.1371/journal.pone.0054784 (PMC3559824; doi:10.1371/journal.pone.0054784)
Supplement: Table S4 — Peak horizontal impact force amplitude: values are expressed as multiples of body weight (x BW); median amplitude (IQR) per species is shown. (DOCX) [file pone.0054784.s007.docx]

Supplementary Table S4: peak horizontal impact force amplitude: values are expressed as multiples of body weight (x BW); median amplitude (IQR) per species is shown.

|  | **Forelimb Walk**  **horizontal**  **impact amplitude (x BW)** | | **Forelimb Slow Run**  **horizontal**  **impact amplitude (x BW)** | | **Hindlimb Walk**  **horizontal**  **impact amplitude (x BW)** | | **Hindlimb Slow Run**  **horizontal**  **impact amplitude (x BW)** | |
| --- | --- | --- | --- | --- | --- | --- | --- | --- |
|  |  |  |  |  |  |  |  |  |
|  |  |  |  |  |  |  |  |  |
| Antelope | 0.31 | (0.10) | 0.22 | (0.09) |  |  |  |  |
| Sheep | 0.15 | (0.13) | 0.40 | (0.24) | 0.15 | (0.05) | 0.30 | (0.14) |
| Pig | 0.11 | (0.01) | 0.04 | (0.09) | 0.11 | (0.02) | 0.11 | (0.04) |
| Addax | 0.23 | (0.16) |  |  | 0.05 | (0.10) |  |  |
| Alpaca | 0.17 | (0.10) | 0.22 | (0.10) | 0.14 | (0.06) | 0.13 | (0.06) |
| Deer | 0.07 | (0.03) | 0.16 | (0.09) | 0.04 | (0.02) | 0.09 | (0.04) |
| Horse | 0.09 | (0.03) | 0.11 | (0.04) | 0.09 | (0.04) | 0.08 | (0.02) |
| Bull | 0.06 | (0.03) |  |  | 0.07 | (0.02) |  |  |
| Dromedary | 0.05 | (0.02) |  |  | 0.04 | (0.01) | 0.07 | (0.01) |
| Giraffe | 0.13 | (0.05) |  |  |  |  |  |  |
| Elephant | 0.06 | (0.03) | 0.21 | (0.06) | 0.05 | (0.02) | 0.04 | (0.01) |
