# Supplementary material for: Size-Related Changes in Foot Impact Mechanics in Hoofed Mammals
Source: PLoS One. 2013 Jan 30;8(1):e54784. doi: 10.1371/journal.pone.0054784 (PMC3559824; doi:10.1371/journal.pone.0054784)
Supplement: Table S5 — Vertical impact velocity– MannWhitney U Test outcomes comparing limb and speed effects. (DOCX) [file pone.0054784.s008.docx]

Supplementary Table S5: vertical impact velocity-- MannWhitney U Test outcomes comparing limb and speed effects. * denotes significant differences between fore- and hind limbs, or between walk and slow run.

|  |  |  |  |  |  |
| --- | --- | --- | --- | --- | --- |
|  |  | **p value** | **Total N** | **Mann-Whitney U** | **Z** |
|  |  |  |  |  |  |
| Forelimb walk versus Hindlimb walk | Sheep | 0.273 | 24 | 53.0 | -1.097 |
|  | Pig | 0.334 | 34 | 116.0 | -0.966 |
|  | Addax | 0.014* | 15 | 7.0 | -2.452 |
|  | Alpaca | 0.541 | 25 | 41.0 | -0.611 |
|  | Deer | <0.001* | 48 | 108.0 | -3.712 |
|  | Horse | 0.819 | 56 | 378.0 | -0.229 |
|  | Bull | <0.001* | 44 | 85.0 | -3.684 |
|  | Dromedary | 0.086 | 32 | 79.0 | -1.715 |
|  | Elephant | 0.409 | 48 | 248.0 | -0.825 |
| Forelimb run versus Hindlimb run | Sheep | 0.121 | 9 | 3.0 | -1.549 |
|  | Pig | 0.009* | 16 | 7.0 | -2.626 |
|  | Alpaca | 0.317 | 8 | 3.0 | -1.000 |
|  | Deer | 0.002* | 20 | 9.0 | -3.038 |
|  | Horse | 0.643 | 13 | 15.0 | -0.468 |
|  | Elephant | 0.564 | 5 | 2.0 | -0.577 |
| Forelimb run versus Forelimb walk | Antelope | 0.006* | 24 | 0.0 | -2.758 |
|  | Sheep | 0.009* | 15 | 0.0 | -2.598 |
|  | Pig | 0.001* | 24 | 12.0 | -3.184 |
|  | Alpaca | 0.114 | 26 | 34.0 | -1.582 |
|  | Deer | 0.001* | 33 | 20.5 | -3.355 |
|  | Horse | 0.026 | 32 | 17.0 | -2.222 |
|  | Elephant | 0.211 | 26 | 11.0 | -1.251 |
| Hindlimb run versus Hindlimb walk | Sheep | 0.001* | 18 | 1.0 | -3.278 |
|  | Pig | 0.579 | 26 | 62.0 | -0.556 |
|  | Alpaca | 0.699 | 7 | 4.0 | -0.387 |
|  | Deer | <0.001* | 35 | 35.5 | -3.577 |
|  | Dromedary | 0.864 | 15 | 12.0 | -0.171 |
|  | Elephant | 0.007* | 27 | 1.0 | -2.700 |
