# Supplementary material for: Size-Related Changes in Foot Impact Mechanics in Hoofed Mammals
Source: PLoS One. 2013 Jan 30;8(1):e54784. doi: 10.1371/journal.pone.0054784 (PMC3559824; doi:10.1371/journal.pone.0054784)
Supplement: Table S6 — Vertical impact velocity: values are expressed in metres per second; median (IQR) per species is shown. (DOCX) [file pone.0054784.s009.docx]

Supplementary Table S6: vertical impact velocity: values are expressed in metres per second; median (IQR) per species is shown.

|  | **Forelimb Walk**  **Impact velocity (ms^-1^)** | | **Forelimb Slow Run**  **Impact velocity (ms^-1^)** | | **Hindlimb Walk**  **Impact velocity (ms^-1^)** | | **Hindlimb Slow Run**  **Impact velocity (ms^-1^)** | |
| --- | --- | --- | --- | --- | --- | --- | --- | --- |
|  |  |  |  |  |  |  |  |  |
|  |  |  |  |  |  |  |  |  |
| Antelope | 0.64 | (0.17) | 1.35 | (0.04) |  |  |  |  |
| Sheep | 0.43 | (0.25) | 1.28 | (0.26) | 0.33 | (0.14) | 0.81 | (0.27) |
| Pig | 0.44 | (0.13) | 0.95 | (0.28) | 0.55 | (0.16) | 0.56 | (0.24) |
| Addax | 0.55 | (0.63) |  |  | 0.28 | (0.16) |  |  |
| Alpaca | 0.34 | (0.24) | 0.22 | (0.14) | 0.40 | (0.17) | 0.54 | (0.27) |
| Deer | 0.66 | (0.25) | 1.56 | (1.13) | 0.48 | (0.18) | 0.98 | (0.19) |
| Horse | 0.41 | (0.41) | 0.79 | (0.05) | 0.62 | (0.77) | 0.77 | (0.21) |
| Bull | 0.62 | (0.23) |  |  | 0.75 | (0.36) |  |  |
| Dromedary | 0.59 | (0.33) |  |  | 0.83 | (0.26) | 0.95 | (0.22) |
| Giraffe | 0.93 | (0.29) |  |  |  |  |  |  |
| Elephant | 1.17 | (0.92) | 2.07 | (0.46) | 1.03 | (0.83) | 1.81 | (0.53) |
