# Supplementary material for: Size-Related Changes in Foot Impact Mechanics in Hoofed Mammals
Source: PLoS One. 2013 Jan 30;8(1):e54784. doi: 10.1371/journal.pone.0054784 (PMC3559824; doi:10.1371/journal.pone.0054784)
Supplement: Table S7 — Horizontal impact velocity– MannWhitney U Test outcomes comparing limb and speed effects. (DOCX) [file pone.0054784.s010.docx]

Supplementary Table S7: horizontal impact velocity-- MannWhitney U Test outcomes comparing limb and speed effects. * denotes significant differences between fore- and hind limbs, or between walk and slow run.

|  |  |  |  |  |  |
| --- | --- | --- | --- | --- | --- |
|  |  | **p value** | **Total N** | **Mann-Whitney U** | **Z** |
|  |  |  |  |  |  |
| Forelimb walk versus Hindlimb walk | Sheep | 0.386 | 24 | 57.0 | -0.866 |
|  | Pig | 0.285 | 34 | 113.0 | -1.070 |
|  | Addax | 0.199 | 15 | 17.0 | -1.285 |
|  | Alpaca | 0.221 | 25 | 32.0 | -1.223 |
|  | Deer | 0.015* | 48 | 170.0 | -2.430 |
|  | Horse | 0.670 | 56 | 366.0 | -0.426 |
|  | Bull | 0.403 | 44 | 206.0 | -0.836 |
|  | Dromedary | 0.118 | 32 | 83.0 | -1.561 |
|  | Elephant | 0.066 | 48 | 199.0 | -1.835 |
| Forelimb run versus Hindlimb run | Sheep | 0.197 | 9 | 4.0 | -1.291 |
|  | Pig | 0.462 | 16 | 25.0 | -0.735 |
|  | Alpaca | 0.046 | 8 | 0.0 | -2.000 |
|  | Deer | 0.019* | 20 | 18.0 | -2.337 |
|  | Horse | 0.005* | 13 | 0.0 | -2.777 |
|  | Elephant | 0.248 | 5 | 1.0 | -1.155 |
| Forelimb run versus Forelimb walk | Antelope | 0.406 | 24 | 22.0 | -0.832 |
|  | Sheep | 0.386 | 15 | 12.0 | -0.866 |
|  | Pig | 0.501 | 24 | 53.0 | -0.674 |
|  | Alpaca | 0.059 | 26 | 29.0 | -1.887 |
|  | Deer | 0.055 | 33 | 54.5 | -1.920 |
|  | Horse | 0.732 | 32 | 50.0 | -0.342 |
|  | Elephant | 0.149 | 26 | 9.0 | -1.443 |
| Hindlimb run versus Hindlimb walk | Sheep | 0.015* | 18 | 10.0 | -2.435 |
|  | Pig | 0.437 | 26 | 58.0 | -0.778 |
|  | Alpaca | 0.053 | 7 | 0.0 | -1.936 |
|  | Deer | 0.005* | 35 | 57.5 | -2.810 |
|  | Horse | <0.001* | 37 | 0.0 | -4.461 |
|  | Dromedary | 0.060 | 15 | 2.0 | -1.878 |
|  | Elephant | 0.037 | 27 | 9.0 | -2.083 |
