# Supplementary material for: Size-Related Changes in Foot Impact Mechanics in Hoofed Mammals
Source: PLoS One. 2013 Jan 30;8(1):e54784. doi: 10.1371/journal.pone.0054784 (PMC3559824; doi:10.1371/journal.pone.0054784)
Supplement: Table S8 — Horizontal impact velocity: values are expressed in metres per second; median (IQR) per species is shown. (DOCX) [file pone.0054784.s011.docx]

Supplementary Table S8: horizontal impact velocity: values are expressed in metres per second; median (IQR) per species is shown.

|  | **Forelimb Walk**  **Impact velocity (ms^-1^)** | | **Forelimb Slow Run**  **Impact velocity (ms^-1^)** | | **Hindlimb Walk**  **Impact velocity (ms^-1^)** | | **Hindlimb Slow Run**  **Impact velocity (ms^-1^)** | |
| --- | --- | --- | --- | --- | --- | --- | --- | --- |
|  |  |  |  |  |  |  |  |  |
|  |  |  |  |  |  |  |  |  |
| Antelope | 0.96 | (0.21) | 1.40 | (0.32) |  |  |  |  |
| Sheep | 0.57 | (2.28) | 1.31 | (0.66) | 1.25 | (0.87) | 2.60 | (1.19) |
| Pig | 0.48 | (0.08) | 0.69 | (0.74) | 0.62 | (0.25) | 1.36 | (2.06) |
| Addax | 1.64 | (1.20) |  |  | 2.63 | (1.03) |  |  |
| Alpaca | 1.27 | (1.34) |  |  | 1.55 | (0.89) | 3.48 | (0.33) |
| Deer | 1.82 | (1.19) | 1.64 | (0.35) | 2.99 | (0.58) | 4.38 | (2.54) |
| Horse | 1.10 | (0.76) | 2.87 | (0.46) | 1.13 | (0.99) | 3.59 | (0.92) |
| Bull | 0.79 | (2.36) | 1.20 | (0.76) | 1.14 | (1.00) |  |  |
| Dromedary | 0.46 | (0.30) |  |  | 0.47 | (0.40) | 1.81 | (0.72) |
| Giraffe | 1.29 | (0.15) |  |  |  |  |  |  |
| Elephant | 0.84 | (0.71) | 1.49 | (0.30) | 0.58 | (0.61) | 1.17 | (0.12) |
