# Supplementary material for: Size-Related Changes in Foot Impact Mechanics in Hoofed Mammals
Source: PLoS One. 2013 Jan 30;8(1):e54784. doi: 10.1371/journal.pone.0054784 (PMC3559824; doi:10.1371/journal.pone.0054784)
Supplement: Table S9 — Impact duration– MannWhitney U Test outcomes comparing limb and speed effects. (DOCX) [file pone.0054784.s012.docx]

Supplementary Table S9: impact duration-- MannWhitney U Test outcomes comparing limb and speed effects. * denotes significant differences between fore- and hind limbs, or between walk and slow run.

|  |  |  |  |  |  |
| --- | --- | --- | --- | --- | --- |
|  |  | **p value** | **Total N** | **Mann-Whitney U** | **Z** |
|  |  |  |  |  |  |
| Forelimb walk versus Hindlimb walk | Sheep | 0.016* | 25 | 34.0 | -2.413 |
|  | Pig | 0.013* | 35 | 77.5 | -2.471 |
|  | Addax | 0.762 | 15 | 25.5 | -0.303 |
|  | Alpaca | 0.065 | 25 | 23.0 | -1.846 |
|  | Deer | 0.009* | 47 | 155.0 | -2.603 |
|  | Horse | 0.057 | 56 | 122.5 | -1.901 |
|  | Bull | 0.004* | 44 | 122.0 | -2.846 |
|  | Dromedary | 0.969 | 32 | 122.5 | -0.039 |
|  | Elephant | 0.001* | 48 | 122.5 | -3.434 |
| Forelimb run versus Hindlimb run | Sheep | 0.433 | 9 | 6.0 | -0.784 |
|  | Pig | 0.228 | 17 | 23.5 | -1.205 |
|  | Alpaca | 0.096 | 8 | 1.0 | -1.667 |
|  | Deer | 0.361 | 20 | 36.5 | -0.914 |
|  | Horse | 0.121 | 14 | 11.0 | -1.552 |
|  | Elephant | 0.275 | 6 | 2.0 | -1.091 |
| Forelimb run versus Forelimb walk | Antelope | 0.327 | 24 | 21.0 | -0.981 |
|  | Sheep | 0.382 | 15 | 12.0 | -0.874 |
|  | Pig | 0.029 | 24 | 28.5 | -2.181 |
|  | Alpaca | 0.314 | 26 | 43.5 | -1.006 |
|  | Deer | 0.322 | 33 | 77.0 | -0.989 |
|  | Horse | 0.256 | 33 | 47.5 | -1.136 |
|  | Elephant | 0.785 | 27 | 32.5 | -0.273 |
| Hindlimb run versus Hindlimb walk | Sheep | 0.691 | 19 | 34.5 | -0.397 |
|  | Pig | 0.022 | 28 | 39.0 | -2.296 |
|  | Alpaca | 1.000 | 7 | 5.0 | 0.000 |
|  | Deer | 0.043 | 34 | 77.0 | -2.027 |
|  | Horse | 0.013* | 37 | 56.0 | -2.495 |
|  | Dromedary | 0.199 | 15 | 5.5 | -1.285 |
|  | Elephant | 0.969 | 27 | 35.5 | -0.039 |
