# Supplementary material for: Size-Related Changes in Foot Impact Mechanics in Hoofed Mammals
Source: PLoS One. 2013 Jan 30;8(1):e54784. doi: 10.1371/journal.pone.0054784 (PMC3559824; doi:10.1371/journal.pone.0054784)
Supplement: Table S10 — Impact duration: values are expressed in milliseconds; median (IQR) per species is shown. (DOCX) [file pone.0054784.s013.docx]

Supplementary Table S10: impact duration: values are expressed in milliseconds; median (IQR) per species is shown.

|  | **Forelimb Walk**  **Impact duration (ms)** | | **Forelimb Slow Run**  **impact duration (ms)** | | **Hindlimb Walk**  **impact duration (ms)** | | **Hindlimb Slow Run**  **impact duration (ms)** | |
| --- | --- | --- | --- | --- | --- | --- | --- | --- |
| Antelope | 20 | (5) | 20 | (3) |  |  |  |  |
| Sheep | 18 | (6) | 18 | (5) | 12 | (11) | 4 | (12) |
| Pig | 26 | (10) | 16 | (10) | 14 | (10) | 11 | (4) |
| Addax | 30 | (10) |  |  | 25 | (5) |  |  |
| Alpaca | 22 | (41) | 41 | (7) | 16 | (2) | 21 | (13) |
| Deer | 20 | (15) | 18 | (6) | 30 | (5) | 23 | (13) |
| Horse | 14 | (14) | 10 | (2) | 20 | (13) | 12 | (2) |
| Bull | 20 | (8) |  |  | 30 | (10) |  |  |
| Dromedary | 35 | (23) |  |  | 50 | (35) | 18 | (8) |
| Giraffe | 20 | (5) |  |  |  |  |  |  |
| Elephant | 55 | (40) | 50 | (25) | 90 | (60) | 80 | (45) |
