# Supplementary material for: Size-Related Changes in Foot Impact Mechanics in Hoofed Mammals
Source: PLoS One. 2013 Jan 30;8(1):e54784. doi: 10.1371/journal.pone.0054784 (PMC3559824; doi:10.1371/journal.pone.0054784)
Supplement: Table S11 — M eff – MannWhitney U Test outcomes comparing limb and speed effects. (DOCX) [file pone.0054784.s014.docx]

Supplementary Table S11: M*_eff_*-- MannWhitney U Test outcomes comparing limb and speed effects. * denotes significant differences between fore- and hind limbs, or between walk and slow run.

|  |  |  |  |  |  |
| --- | --- | --- | --- | --- | --- |
|  |  | **p value** | **Total N** | **Mann-Whitney U** | **Z** |
|  |  |  |  |  |  |
| Forelimb walk versus Hindlimb walk | Sheep | 0.003* | 24 | 20.0 | -3.002 |
|  | Pig | 0.945 | 34 | 142.0 | -0.069 |
|  | Addax | 0.908 | 15 | 27.0 | -0.116 |
|  | Alpaca | 0.103 | 25 | 26.0 | -1.630 |
|  | Deer | 0.406 | 47 | 236.0 | -0.831 |
|  | Horse | 0.806 | 56 | 377.0 | -0.246 |
|  | Bull | 0.897 | 44 | 236.0 | -0.129 |
|  | Dromedary | 0.103 | 32 | 81.0 | -1.631 |
|  | Elephant | 0.002 | 45 | 113.0 | -3.162 |
| Forelimb run versus Hindlimb run | Sheep | 0.439 | 9 | 6.0 | -0.775 |
|  | Pig | 0.753 | 16 | 29.0 | -0.315 |
|  | Alpaca | 0.046 | 8 | 0.0 | -2.000 |
|  | Deer | 0.396 | 20 | 37.0 | -0.849 |
|  | Horse | 0.014* | 13 | 2.0 | -2.469 |
|  | Elephant | 0.564 | 5 | 2.0 | -0.577 |
| Forelimb run versus Forelimb walk | Antelope | 0.383 | 23 | 13.0 | -0.873 |
|  | Sheep | 0.149 | 15 | 8.0 | -1.443 |
|  | Pig | 0.017* | 24 | 25.0 | -2.388 |
|  | Alpaca | 0.068 | 26 | 30.0 | -1.826 |
|  | Deer | 0.966 | 33 | 99.0 | -0.042 |
|  | Horse | 1.000 | 32 | 56.0 | 0.000 |
|  | Elephant | 0.050 | 23 | 3.0 | -1.964 |
| Hindlimb run versus Hindlimb walk | Sheep | 0.025 | 18 | 12.0 | -2.248 |
|  | Pig | 0.035 | 26 | 34.0 | -2.111 |
|  | Alpaca | 1.000 | 7 | 5.0 | 0.000 |
|  | Deer | 0.589 | 34 | 117.0 | -0.541 |
|  | Horse | 0.750 | 37 | 117.0 | -0.319 |
|  | Dromedary | 0.396 | 15 | 8.0 | -0.849 |
|  | Elephant | 0.537 | 27 | 28.0 | -0.617 |
