# Supplementary material for: Size-Related Changes in Foot Impact Mechanics in Hoofed Mammals
Source: PLoS One. 2013 Jan 30;8(1):e54784. doi: 10.1371/journal.pone.0054784 (PMC3559824; doi:10.1371/journal.pone.0054784)
Supplement: Table S12 — M eff values are expressed as a percentage of body weight; median (IQR) per species is shown. (DOCX) [file pone.0054784.s015.docx]

Supplementary Table S12: M*_eff_* values are expressed as a percentage of body weight; median (IQR) per species is shown.

|  | **Forelimb Walk**  **M*_eff_* (% BW)** | | **Forelimb Slow Run**  **M*_eff_* (% BW)** | | **Hindlimb Walk**  **M*_eff_* (% BW)** | | | **Hindlimb Slow Run**  **M*_eff_* (% BW)** | |
| --- | --- | --- | --- | --- | --- | --- | --- | --- | --- |
|  |  |  |  |  |  |  |  |  |  |
|  |  |  |  |  |  |  |  |  |  |
| Antelope | 4.21 | (3.28) | 4.71 | (0.67) |  |  | |  |  |
| Sheep | 0.63 | (1.15) | 2.00 | (0.78) | 0.06 | | (0.12) | 0.23 | (2.21) |
| Pig | 0.21 | (0.75) | 1.24 | (1.57) | 0.16 | | (0.49) | 0.60 | (0.17) |
| Addax | 4.93 | (3.34) |  |  | 4.87 | | (5.04) |  |  |
| Alpaca | 0.58 | (2.64) | 1.93 | (0.89) | 0.35 | | (0.19) | 0.41 | (0.29) |
| Deer | 1.61 | (0.66) | 1.63 | (0.75) | 1.78 | | (0.69) | 1.95 | (1.26) |
| Horse | 0.39 | (0.98) | 0.53 | (0.05) | 0.11 | | (3.46) | 0.19 | (0.07) |
| Bull | 0.91 | (1.16) |  |  | 1.00 | | (0.62) |  |  |
| Dromedary | 0.68 | (1.94) |  |  | 0.56 | | (0.94) | 1.07 | (0.30) |
| Giraffe | 3.34 | (1.57) |  |  |  | |  |  |  |
| Elephant | 0.45 | (0.30) | 1.40 | (0.44) | 0.82 | | (0.93) | 0.45 | (1.03) |
