# Supplementary material for: Size-Related Changes in Foot Impact Mechanics in Hoofed Mammals
Source: PLoS One. 2013 Jan 30;8(1):e54784. doi: 10.1371/journal.pone.0054784 (PMC3559824; doi:10.1371/journal.pone.0054784)
Supplement: Table S13 — Maximum average loading rate (calculated over a window of 0.5% stance during the initial 25% stance)– MannWhitney U Test outcomes comparing limb and speed effects. (DOCX) [file pone.0054784.s016.docx]

Supplementary Table S13: maximum average loading rate (calculated over a window of 0.5% stance during the initial 25% stance)-- MannWhitney U Test outcomes comparing limb and speed effects. * denotes significant differences between fore- and hind limbs, or between walk and slow run.

|  |  |  |  |  |  |
| --- | --- | --- | --- | --- | --- |
|  |  | **p value** | **Total N** | **Mann-Whitney U** | **Z** |
|  |  |  |  |  |  |
| Forelimb walk versus Hindlimb walk | Sheep | 0.003* | 25 | 24.0 | -2.941 |
|  | Pig | 0.619 | 35 | 137.0 | -0.497 |
|  | Addax | 0.001* | 17 | 0.0 | -3.466 |
|  | Alpaca | 0.861 | 27 | 60.0 | -0.176 |
|  | Deer | <0.001* | 47 | 87.5 | -3.998 |
|  | Horse | <0.001* | 56 | 113.5 | -4.564 |
|  | Bull | 0.002* | 44 | 110.0 | -3.090 |
|  | Dromedary | 0.009* | 32 | 55.0 | -2.628 |
|  | Giraffe | 0.127 | 8 | 0.0 | -1.528 |
|  | Elephant | 0.068 | 43 | 155.0 | -1.826 |
| Forelimb run versus Hindlimb run | Sheep | 0.068 | 9 | 2.0 | -1.823 |
|  | Pig | 0.075 | 17 | 17.5 | -1.782 |
|  | Alpaca | 0.505 | 8 | 4.0 | -0.667 |
|  | Deer | 0.021 | 20 | 18.0 | -2.316 |
|  | Horse | 0.016* | 14 | 4.5 | -2.408 |
|  | Dromedary | 0.221 | 3 | 0.0 | -1.225 |
|  | Elephant | 0.827 | 6 | 4.0 | -0.218 |
| Forelimb run versus Forelimb walk | Antelope | 0.006* | 24 | 0.0 | -2.750 |
|  | Sheep | 0.009* | 15 | 0.0 | -2.614 |
|  | Pig | <0.001* | 24 | 0.0 | -3.515 |
|  | Alpaca | <0.001* | 27 | 0.0 | -3.686 |
|  | Deer | <0.001* | 33 | 0.0 | -4.203 |
|  | Horse | <0.001* | 33 | 0.0 | -3.515 |
|  | Dromedary | 0.099 | 20 | 0.0 | -1.648 |
|  | Elephant | 0.160 | 26 | 17.0 | -1.405 |
| Hindlimb run versus Hindlimb walk | Sheep | 0.001 | 19 | 0.0 | -3.424 |
|  | Pig | <0.001* | 28 | 0.0 | -4.207 |
|  | Alpaca | 0.046 | 8 | 0.0 | -2.000 |
|  | Deer | <0.001* | 34 | 0.0 | -4.758 |
|  | Horse | <0.001* | 37 | 0.0 | -4.461 |
|  | Dromedary | 0.027 | 15 | 0.0 | -2.208 |
|  | Elephant | 0.068 | 23 | 10.0 | -1.826 |
