# Supplementary material for: Size-Related Changes in Foot Impact Mechanics in Hoofed Mammals
Source: PLoS One. 2013 Jan 30;8(1):e54784. doi: 10.1371/journal.pone.0054784 (PMC3559824; doi:10.1371/journal.pone.0054784)
Supplement: Table S14 — Maximum average loading rate (calculated over a window of 0.5% stance during the initial 25% stance): values are expressed in body weights per seconds (Mb s−1); median loading rate (IQR) per species is shown. (DOCX) [file pone.0054784.s017.docx]

Supplementary Table S14: maximum average loading rate (calculated over a window of 0.5% stance during the initial 25% stance): values are expressed in body weights per seconds

(M_b_ s^-1^); median loading rate (IQR) per species is shown.

|  | **Forelimb Walk**  **Max. Loading Rate (M_b_ s^-1^)** | | **Forelimb Slow Run**  **Max. Loading Rate (M_b_ s^-1^)** | | **Hindlimb Walk**  **Max. Loading Rate (M_b_ s^-1^)** | | **Hindlimb Slow Run**  **Max. Loading Rate (M_b_ s^-1^)** | |
| --- | --- | --- | --- | --- | --- | --- | --- | --- |
| Antelope | 4359.90 | (2030.40) | 15828.00 | (1458.50) |  |  |  |  |
| Sheep | 5049.80 | (5203.64) | 15379.00 | (7136.30) | 64.67 | (31.55) | 742.44 | (11028.59) |
| Pig | 52.37 | (2621.35) | 10885.00 | (10981.26) | 60.93 | (2087.09) | 1008.70 | (8620.16) |
| Addax | 3948.30 | (776.50) |  |  | 2371.80 | (535.63) |  |  |
| Alpaca | 226.88 | (3549.80) | 8726.95 | (3228.03) | 163.86 | (88.13) | 6635.44 | (5652.57) |
| Deer | 1295.90 | (334.60) | 4161.40 | (1492.98) | 914.14 | (431.71) | 3575.80 | (782.75) |
| Horse | 812.38 | (1655.01) | 325.14 | (3.33) | 29.59 | (1251.67) | 239.54 | (25.82) |
| Bull | 944.38 | (266.14) |  |  | 703.43 | (306.14) |  |  |
| Dromedary | 607.70 | (134.98) |  |  | 454.77 | (48.41) | 2103.75 | (297.15) |
| Giraffe | 1035.80 | (64.48) |  |  |  |  |  |  |
| Elephant | 246.80 | (465.91) | 313.16 | (2108.39) | 191.41 | (519.25) | 315.90 | (1496.69) |
