# Supplementary material for: Size-Related Changes in Foot Impact Mechanics in Hoofed Mammals
Source: PLoS One. 2013 Jan 30;8(1):e54784. doi: 10.1371/journal.pone.0054784 (PMC3559824; doi:10.1371/journal.pone.0054784)
Supplement: Table S15 — Maximum instantaneous loading rate– Mann Whitney U Test outcomes comparing limb and speed effects. (DOCX) [file pone.0054784.s018.docx]

Supplementary Table S15: maximum instantaneous loading rate-- Mann Whitney U Test outcomes comparing limb and speed effects. * denotes significant differences between fore- and hind limbs, or between walk and slow run.

|  |  |  |  |  |  |
| --- | --- | --- | --- | --- | --- |
|  |  | **p value** | **Total N** | **Mann-Whitney U** | **Z** |
|  |  |  |  |  |  |
| Forelimb walk versus Hindlimb walk | Sheep | 0.355 | 25 | 61.0 | -0.925 |
|  | Pig | 0.020* | 35 | 82.0 | -2.318 |
|  | Addax | 0.001* | 17 | 0.0 | -3.464 |
|  | Alpaca | 0.188 | 27 | 40.5 | -1.316 |
|  | Deer | <0.001* | 47 | 50.0 | -4.797 |
|  | Horse | 0.004* | 56 | 215.0 | -2.902 |
|  | Bull | 0.254 | 44 | 193.0 | -1.140 |
|  | Dromedary | 0.478 | 32 | 105.0 | -0.710 |
|  | Giraffe | 0.127 | 8 | 0.0 | -1.528 |
|  | Elephant | 0.543 | 43 | 205.0 | -0.609 |
| Forelimb run versus Hindlimb run | Sheep | 0.439 | 9 | 6.0 | -0.775 |
|  | Pig | 0.178 | 17 | 22.0 | -1.347 |
|  | Alpaca | 0.317 | 8 | 3.0 | -1.000 |
|  | Deer | 0.009* | 20 | 14.0 | -2.623 |
|  | Horse | 0.082 | 14 | 9.50 | -1.737 |
|  | Dromedary | 0.221 | 3 | 0.0 | -1.225 |
|  | Elephant | 0.513 | 6 | 3.0 | -0.655 |
| Forelimb run versus Forelimb walk | Antelope | 0.106 | 24 | 13.0 | -1.615 |
|  | Sheep | 0.009* | 15 | 0.0 | -2.598 |
|  | Pig | <0.001* | 24 | 4.0 | -3.674 |
|  | Alpaca | 0.008* | 27 | 18.0 | -2.633 |
|  | Deer | 0.004* | 33 | 31.0 | -2.899 |
|  | Horse | 0.001* | 33 | 1.0 | -3.466 |
|  | Dromedary | 0.099 | 20 | 0.0 | -1.648 |
|  | Elephant | 0.049 | 26 | 10.0 | -1.967 |
| Hindlimb run versus Hindlimb walk | Sheep | 0.001* | 19 | 0.0 | -3.421 |
|  | Pig | <0.001* | 28 | 7.0 | -3.862 |
|  | Alpaca | 0.046 | 8 | 0.0 | -2.000 |
|  | Deer | <0.001* | 34 | 15.0 | -4.216 |
|  | Horse | 0.229 | 37 | 92.0 | -1.204 |
|  | Dromedary | 0.027 | 15 | 0.0 | -2.208 |
|  | Elephant | 0.100 | 23 | 12.0 | -1.644 |
