# Supplementary material for: Size-Related Changes in Foot Impact Mechanics in Hoofed Mammals
Source: PLoS One. 2013 Jan 30;8(1):e54784. doi: 10.1371/journal.pone.0054784 (PMC3559824; doi:10.1371/journal.pone.0054784)
Supplement: Table S16 — Maximum instantaneous loading rate: values are expressed in body weights per second (Mb s−1); median loading rate (IQR) per species is shown. (DOCX) [file pone.0054784.s019.docx]

Supplementary Table S16: maximum instantaneous loading rate: values are expressed in body weights per second (M_b_ s^-1^); median loading rate (IQR) per species is shown.

|  | **Forelimb Walk**  **Max. Inst. L. Rate (M_b_ s^-1^)** | | **Forelimb Slow Run**  **Max. Inst. L. Rate (M_b_ s^-1^)** | | **Hindlimb Walk**  **Max. Inst. L. Rate (M_b_ s^-1^)** | | **Hindlimb Slow Run**  **Max. Inst. L. Rate (M_b_ s^-1^)** | |
| --- | --- | --- | --- | --- | --- | --- | --- | --- |
|  |  |  |  |  |  |  |  |  |
|  |  |  |  |  |  |  |  |  |
| Antelope | 35.82 | (27.63) | 81.59 | (14.13) |  |  |  |  |
| Sheep | 39.34 | (22.67) | 115.97 | (50.52) | 33.74 | (9.58) | 121.17 | (20.38) |
| Pig | 18.49 | (9.38) | 59.11 | (32.52) | 27.47 | (8.80) | 83.60 | (47.93) |
| Addax | 29.27 | (16.13) |  |  | 16.18 | (3.05) |  |  |
| Alpaca | 21.98 | (27.09) | 46.40 | (12.55) | 36.47 | (11.09) | 64.37 | (18.00) |
| Deer | 22.25 | (17.99) | 46.89 | (33.09) | 10.74 | (0.95) | 23.69 | (5.51) |
| Horse | 19.74 | (10.17) | 49.26 | (53.40) | 36.18 | (27.73) | 35.13 | (14.24) |
| Bull | 6.52 | (3.31) |  |  | 5.27 | (2.40) |  |  |
| Dromedary | 2.67 | (1.16) |  |  | 2.20 | (1.26) | 10.23 | (4.17) |
| Giraffe | 23.95 | (6.52) |  |  |  |  |  |  |
| Elephant | 6.32 | (3.27) | 18.61 | (8.48) | 5.67 | (5.71) | 12.56 | (5.03) |
