# Supplementary material for: Size-Related Changes in Foot Impact Mechanics in Hoofed Mammals
Source: PLoS One. 2013 Jan 30;8(1):e54784. doi: 10.1371/journal.pone.0054784 (PMC3559824; doi:10.1371/journal.pone.0054784)
Supplement: Table S17 — Peak vertical ground reaction force (GRF) amplitude– MannWhitney U Test outcomes comparing limb and speed effects. (DOCX) [file pone.0054784.s020.docx]

Supplementary Table S17: peak vertical ground reaction force (GRF) amplitude-- MannWhitney U Test outcomes comparing limb and speed effects. * denotes significant differences between fore- and hind limbs, or between walk and slow run.

|  |  |  |  |  |  |
| --- | --- | --- | --- | --- | --- |
|  |  | **p value** | **Total N** | **Mann-Whitney U** | **Z** |
|  |  |  |  |  |  |
| Forelimb walk versus Hindlimb walk | Sheep | 0.034 | 25 | 39.0 | -2.122 |
|  | Pig | 0.029 | 35 | 86.0 | -2.185 |
|  | Addax | 0.001* | 15 | 0.0 | -3.240 |
|  | Alpaca | 0.164 | 25 | 29.5 | -1.393 |
|  | Deer | <0.001* | 48 | 40.0 | -5.108 |
|  | Horse | <0.001* | 56 | 149.0 | -3.982 |
|  | Bull | <0.001* | 44 | 1.0 | -5.651 |
|  | Dromedary | <0.001* | 32 | 4.0 | -4.585 |
|  | Elephant | <0.001* | 48 | 40.0 | -5.114 |
| Forelimb run versus Hindlimb run | Sheep | 0.020* | 9 | 0.0 | -2.334 |
|  | Pig | 0.441 | 17 | 28.0 | -0.770 |
|  | Alpaca | 0.317 | 8 | 3.0 | -1.000 |
|  | Deer | 0.007* | 20 | 13.0 | -2.700 |
|  | Horse | 0.011* | 14 | 3.5 | -2.536 |
|  | Elephant | 0.050 | 6 | 0.0 | -1.964 |
| Forelimb run versus Forelimb walk | Antelope | 0.006* | 24 | 0.0 | -2.750 |
|  | Sheep | 0.009* | 15 | 0.0 | -2.598 |
|  | Pig | <0.001* | 24 | 1.0 | -3.858 |
|  | Alpaca | <0.001* | 26 | 0.0 | -3.652 |
|  | Deer | <0.001* | 33 | 0.0 | -4.201 |
|  | Horse | 0.003* | 33 | 11.0 | -2.962 |
|  | Elephant | 0.007* | 27 | 1.0 | -2.701 |
| Hindlimb run versus Hindlimb walk | Sheep | 0.001* | 19 | 0.0 | -3.424 |
|  | Pig | <0.001* | 28 | 0.0 | -4.206 |
|  | Alpaca | 0.245 | 7 | 2.0 | -1.162 |
|  | Deer | <0.001* | 35 | 4.0 | -4.657 |
|  | Horse | <0.001* | 37 | 16.0 | -3.894 |
|  | Dromedary | 0.027 | 15 | 0.0 | -2.208 |
|  | Elephant | 0.054 | 27 | 11.0 | -1.929 |
