# Supplementary material for: Size-Related Changes in Foot Impact Mechanics in Hoofed Mammals
Source: PLoS One. 2013 Jan 30;8(1):e54784. doi: 10.1371/journal.pone.0054784 (PMC3559824; doi:10.1371/journal.pone.0054784)
Supplement: Table S18 — Peak vertical ground reaction force (GRF) amplitude: values are expressed as multiples of body weight; median amplitude (IQR) per species is shown. (DOCX) [file pone.0054784.s021.docx]

Supplementary Table S18: peak vertical ground reaction force (GRF) amplitude: values are expressed as multiples of body weight; median amplitude (IQR) per species is shown.

|  | **Forelimb Walk**  **GRF amplitude (xBW)** | | **Forelimb Slow Run**  **GRF amplitude (xBW)** | | **Hindlimb Walk**  **GRF amplitude (xBW)** | | **Hindlimb Slow Run**  **GRF amplitude (xBW)** | |
| --- | --- | --- | --- | --- | --- | --- | --- | --- |
|  |  |  |  |  |  |  |  |  |
|  |  |  |  |  |  |  |  |  |
| Antelope | 1.37 | (0.53) | 2.87 | (0.63) |  |  |  |  |
| Sheep | 1.36 | (0.38) | 2.35 | (0.15) | 0.80 | (0.02) | 1.41 | (0.22) |
| Pig | 0.59 | (0.12) | 0.95 | (0.38) | 0.52 | (0.05) | 0.86 | (0.10) |
| Addax | 1.50 | (0.22) |  |  | 0.90 | (0.12) |  |  |
| Alpaca | 0.80 | (0.44) | 1.32 | (0.16) | 0.55 | (0.48) | 1.03 | (0.34) |
| Deer | 0.37 | (0.06) | 0.61 | (0.17) | 0.28 | (0.05) | 0.47 | (0.11) |
| Horse | 0.49 | (0.17) | 0.76 | (0.01) | 0.46 | (0.16) | 0.56 | (0.04) |
| Bull | 0.45 | (0.07) |  |  | 0.30 | (0.05) |  |  |
| Dromedary | 0.37 | (0.05) |  |  | 0.23 | (0.01) | 0.39 | (0.04) |
| Giraffe | 1.11 | (0.25) |  |  |  |  |  |  |
| Elephant | 0.65 | (0.04) | 0.75 | (0.08) | 0.42 | (0.08) | 0.60 | (0.10) |
