# Supplementary material for: Size-Related Changes in Foot Impact Mechanics in Hoofed Mammals
Source: PLoS One. 2013 Jan 30;8(1):e54784. doi: 10.1371/journal.pone.0054784 (PMC3559824; doi:10.1371/journal.pone.0054784)
Supplement: Table S19 — Vertical impact impulse– MannWhitney U Test outcomes comparing limb and speed effects. (DOCX) [file pone.0054784.s022.docx]

Supplementary Table S19: vertical impact impulse-- MannWhitney U Test outcomes comparing limb and speed effects. * denotes significant differences between fore- and hind limbs, or between walk and slow run.

|  |  |  |  |  |  |
| --- | --- | --- | --- | --- | --- |
|  |  | **p value** | **Total N** | **Mann-Whitney U** | **Z** |
|  |  |  |  |  |  |
| Forelimb walk versus Hindlimb walk | Sheep | 0.034 | 35 | 39.0 | -2.121 |
|  | Pig | 0.012* | 35 | 76.0 | -2.517 |
|  | Addax | 0.011* | 15 | 6.0 | -2.546 |
|  | Alpaca | 0.324 | 25 | 35.5 | -0.985 |
|  | Deer | 0.070 | 47 | 190.0 | -1.812 |
|  | Horse | 0.413 | 56 | 342.0 | -0.819 |
|  | Bull | 0.102 | 44 | 172.0 | -1.633 |
|  | Dromedary | 0.432 | 32 | 103.0 | -0.787 |
|  | Elephant | <0.001* | 45 | 68.0 | -4.186 |
| Forelimb run versus Hindlimb run | Sheep | 0.439 | 9 | 6.0 | -0.775 |
|  | Pig | 0.345 | 16 | 23.0 | -0.945 |
|  | Alpaca | 0.046 | 8 | 0.0 | -2.000 |
|  | Deer | 0.017* | 20 | 17.0 | -2.392 |
|  | Horse | 0.083 | 14 | 9.5 | -1.735 |
|  | Elephant | 0.513 | 6 | 3.0 | -0.655 |
| Forelimb run versus Forelimb walk | Antelope | 0.089 | 24 | 12.0 | -1.702 |
|  | Sheep | 0.043 | 15 | 4.0 | -2.021 |
|  | Pig | 0.582 | 24 | 55.0 | -0.551 |
|  | Alpaca | 0.670 | 26 | 53.0 | -0.426 |
|  | Deer | <0.001* | 33 | 13.0 | -3.655 |
|  | Horse | 0.056 | 33 | 32.0 | -1.908 |
|  | Elephant | 0.013* | 24 | 3.0 | -2.488 |
| Hindlimb run versus Hindlimb walk | Sheep | 0.483 | 19 | 31.0 | -0.702 |
|  | Pig | 0.671 | 27 | 68.0 | -0.425 |
|  | Alpaca | 0.053 | 7 | 0.0 | -1.936 |
|  | Deer | 0.001* | 34 | 37.0 | -3.424 |
|  | Horse | 0.066 | 37 | 74.0 | -1.841 |
|  | Dromedary | 0.865 | 15 | 12.0 | -0.170 |
|  | Elephant | 0.396 | 27 | 25.0 | -0.849 |
