# Supplementary material for: Size-Related Changes in Foot Impact Mechanics in Hoofed Mammals
Source: PLoS One. 2013 Jan 30;8(1):e54784. doi: 10.1371/journal.pone.0054784 (PMC3559824; doi:10.1371/journal.pone.0054784)
Supplement: Table S20 — Vertical impact impulse: values are expressed as percentage of body weight per second (%BW s): median impact impulse (IQR) per species is shown. (DOCX) [file pone.0054784.s023.docx]

Supplementary Table S20: vertical impact impulse: values are expressed as percentage of body weight per second (%BW s): median impact impulse (IQR) per species is shown.

|  | **Forelimb Walk**  **Vertical Impact Impulse (%BWs)** | | **Forelimb Slow Run**  **Vertical Impact Impulse (%BWs)** | | **Hindlimb Walk**  **Vertical Impact Impulse (%BWs)** | | **Hindlimb Slow Run**  **Vertical Impact Impulse (%BWs)** | |
| --- | --- | --- | --- | --- | --- | --- | --- | --- |
|  |  |  |  |  |  |  |  |  |
|  |  |  |  |  |  |  |  |  |
| Antelope | 3.53 | (2.61) | 7.29 | (1.46) |  |  |  |  |
| Sheep | 1.10 | (1.14) | 2.55 | (0.28) | 0.34 | (0.32) | 0.32 | (2.51) |
| Pig | 6.57 | (2.88) | 1.18 | (1.26) | 0.94 | (0.22) | 1.04 | (0.54) |
| Addax | 1.45 | (0.99) |  |  | 2.45 | (1.56) |  |  |
| Alpaca | 2.55 | (1.96) | 1.40 | (3.95) | 1.73 | (0.14) | 0.44 | (0.30) |
| Deer | 1.50 | (0.44) | 3.48 | (1.28) | 1.40 | (0.50) | 2.28 | (0.94) |
| Horse | 0.69 | (0.17) | 1.09 | (0.20) | 0.66 | (0.89) | 0.41 | (0.08) |
| Bull | 0.83 | (0.91) |  |  | 1.16 | (0.95) |  |  |
| Dromedary | 0.83 | (2.65) |  |  | 1.32 | (1.57) | 1.16 | (0.18) |
| Giraffe | 3.75 | (1.44) |  |  |  |  |  |  |
| Elephant | 2.17 | (2.63) | 11.38 | (2.22) | 7.04 | (5.59) | 2.39 | (0.21) |
