# Supplementary material for: Size-Related Changes in Foot Impact Mechanics in Hoofed Mammals
Source: PLoS One. 2013 Jan 30;8(1):e54784. doi: 10.1371/journal.pone.0054784 (PMC3559824; doi:10.1371/journal.pone.0054784)
Supplement: Table S21 — (abs) horizontal impact impulse– MannWhitney U Test outcomes comparing limb and speed effects. (DOCX) [file pone.0054784.s024.docx]

Supplementary Table S21: (abs) horizontal impact impulse-- MannWhitney U Test outcomes comparing limb and speed effects. * denotes significant differences between fore- and hind limbs, or between walk and slow run.

|  |  |  |  |  |  |
| --- | --- | --- | --- | --- | --- |
|  |  | **p value** | **Total N** | **Mann-Whitney U** | **Z** |
|  |  |  |  |  |  |
| Forelimb walk versus Hindlimb walk | Sheep | 0.001* | 25 | 18.0 | -3.264 |
|  | Pig | <0.001* | 35 | 36.0 | -3.841 |
|  | Addax | 0.001* | 15 | 0.0 | -3.240 |
|  | Alpaca | 0.025 | 25 | 17.0 | -2.242 |
|  | Deer | <0.001* | 47 | 0.0 | -5.863 |
|  | Horse | <0.001* | 51 | 64.0 | -4.884 |
|  | Bull | <0.001* | 44 | 37.0 | -4.805 |
|  | Dromedary | <0.001* | 32 | 24.0 | -3.818 |
|  | Elephant | <0.001* | 45 | 86.0 | -3.777 |
| Forelimb run versus Hindlimb run | Sheep | 0.439 | 9 | 3.0 | -0.775 |
|  | Pig | 0.773 | 17 | 33.0 | -0.289 |
|  | Alpaca | 0.046 | 8 | 0.0 | -2.000 |
|  | Deer | 0.440 | 20 | 38.0 | -0.772 |
|  | Horse | 0.059 | 11 | 1.0 | -1.886 |
|  | Elephant | 0.827 | 6 | 4.0 | -0.218 |
| Forelimb run versus Forelimb walk | Antelope | 0.106 | 24 | 13.0 | -1.615 |
|  | Sheep | 0.773 | 15 | 16.0 | -0.289 |
|  | Pig | 0.001* | 24 | 10.0 | -3.307 |
|  | Alpaca | 0.301 | 26 | 43.0 | -1.035 |
|  | Deer | <0.001* | 33 | 1.0 | -4.159 |
|  | Horse | 0.021 | 25 | 0.0 | -2.304 |
|  | Elephant | 0.032 | 24 | 7.0 | -2.139 |
| Hindlimb run versus Hindlimb walk | Sheep | 0.930 | 19 | 38.0 | -0.088 |
|  | Pig | 0.605 | 28 | 75.0 | -0.517 |
|  | Alpaca | 0.053 | 7 | 0.0 | -1.936 |
|  | Deer | 0.047 | 34 | 77.0 | -1.982 |
|  | Horse | 0.416 | 37 | 103.0 | -0.814 |
|  | Dromedary | 0.089 | 15 | 3.0 | -1.698 |
|  | Elephant | 0.537 | 27 | 28.0 | -0.617 |
