# Supplementary material for: Size-Related Changes in Foot Impact Mechanics in Hoofed Mammals
Source: PLoS One. 2013 Jan 30;8(1):e54784. doi: 10.1371/journal.pone.0054784 (PMC3559824; doi:10.1371/journal.pone.0054784)
Supplement: Table S22 — (abs) horizontal impact impulse: values are expressed in percentage bodyweight per second (%BW s); median impact impulse (IQR) per species is shown. (DOCX) [file pone.0054784.s025.docx]

Supplementary Table S22: (abs) horizontal impact impulse: values are expressed in percentage bodyweight per second (%BW s); median impact impulse (IQR) per species is shown.

|  | **Forelimb Walk**  **(abs) Horizontal Impact Impulse (%BWs)** | | **Forelimb Slow Run**  **(abs) Horizontal Impact Impulse (%BWs)** | | **Hindlimb Walk**  **(abs) Horizontal Impact Impulse (%BWs)** | | **Hindlimb Slow Run**  **(abs) Horizontal Impact Impulse (%BWs)** | |
| --- | --- | --- | --- | --- | --- | --- | --- | --- |
|  |  |  |  |  |  |  |  |  |
|  |  |  |  |  |  |  |  |  |
| Antelope | 0.0353 | (0.0261) | 0.0137 | (0.0071) | 0.0010 | (0.0009) |  |  |
| Sheep | 0.0110 | (0.0114) | 0.0093 | (0.0017) | 0.0034 | (0.0019) | 0.0008 | (0.0129) |
| Pig | 0.0145 | (0.0099) | 0.0016 | (0.0036) | 0.0027 | (0.0019) | 0.0034 | (0.0029) |
| Addax | 0.0657 | (0.0288) |  |  | 0.0107 | (0.0007) |  |  |
| Alpaca | 0.0255 | (0.0196) | 0.0075 | (0.0200) | 0.0015 | (0.0007) | 0.0022 | (0.0019) |
| Deer | 0.0150 | (0.0044) | 0.0035 | (0.0040) | 0.0025 | (0.0018) | 0.0026 | (0.0020) |
| Horse | 0.0075 | (0.0069) | 0.0002 | (0.0002) | 0.0012 | (0.0009) | 0.0020 | (0.0008) |
| Bull | 0.0083 | (0.0091) |  |  | 0.0030 | (0.0037) |  |  |
| Dromedary | 0.0083 | (0.0265) |  |  | 0.0010 | (0.0009) | 0.0061 | (0.0008) |
| Giraffe | 0.0375 | (0.0144) |  |  |  |  |  |  |
| Elephant | 0.0217 | (0.0263) | 0.0096 | (0.0049) | 0.0063 | (0.0095) | 0.0126 | (0.0157) |
