# Supplementary material for: Size-Related Changes in Foot Impact Mechanics in Hoofed Mammals
Source: PLoS One. 2013 Jan 30;8(1):e54784. doi: 10.1371/journal.pone.0054784 (PMC3559824; doi:10.1371/journal.pone.0054784)
Supplement: Table S23 — Total decelerative impulse– MannWhitney U Test outcomes comparing limb and speed effects. (DOCX) [file pone.0054784.s026.docx]

Supplementary Table S23: total decelerative impulse-- MannWhitney U Test outcomes comparing limb and speed effects. * denotes significant differences between fore- and hind limbs, or between walk and slow run.

|  |  |  |  |  |  |
| --- | --- | --- | --- | --- | --- |
|  |  | **p value** | **Total N** | **Mann-Whitney U** | **Z** |
|  |  |  |  |  |  |
| Forelimb walk versus Hindlimb walk | Sheep | 0.009* | 25 | 30.0 | -2.611 |
|  | Pig | 0.371 | 35 | 125.0 | -0.894 |
|  | Addax | 0.012* | 17 | 10.0 | -2.502 |
|  | Deer | 0.845 | 48 | 278.0 | -0.196 |
|  | Horse | 0.007* | 56 | 228.0 | -2.687 |
|  | Bull | 0.445 | 44 | 209.0 | -0.764 |
|  | Dromedary | <0.001* | 32 | 4.0 | -4.585 |
|  | Elephant | <0.001* | 43 | 36.0 | -4.724 |
| Forelimb run versus Hindlimb run | Sheep | 0.796 | 9 | 8.0 | -0.258 |
|  | Pig | 0.034 | 17 | 14.0 | -2.117 |
|  | Deer | 0.003* | 20 | 9.0 | -3.009 |
|  | Horse | 0.003* | 14 | 0.0 | -3.000 |
|  | Elephant | 0.050 | 6 | 0.0 | -1.964 |
| Forelimb run versus Forelimb walk | Antelope | 0.008* | 24 | 1.0 | -2.663 |
|  | Sheep | 0.021 | 15 | 2.0 | -2.309 |
|  | Pig | <0.001* | 24 | 1.0 | -3.858 |
|  | Deer | 0.153 | 33 | 66.0 | -1.428 |
|  | Horse | 0.021 | 33 | 24.0 | -2.310 |
|  | Elephant | 0.547 | 26 | 27.0 | -0.602 |
| Hindlimb run versus Hindlimb walk | Sheep | 0.861 | 19 | 37.0 | -0.175 |
|  | Pig | <0.001* | 28 | 10.0 | -3.714 |
|  | Deer | 0.244 | 35 | 104.5 | -1.164 |
|  | Horse | 0.002* | 37 | 37.0 | -3.151 |
|  | Dromedary | 0.229 | 15 | 5.0 | -1.359 |
|  | Elephant | 0.584 | 23 | 24.0 | -0.548 |
