# Supplementary material for: Size-Related Changes in Foot Impact Mechanics in Hoofed Mammals
Source: PLoS One. 2013 Jan 30;8(1):e54784. doi: 10.1371/journal.pone.0054784 (PMC3559824; doi:10.1371/journal.pone.0054784)
Supplement: Table S24 — Total decelerative impulse– values are expressed in percentage bodyweight per second (%BW s); median impact impulse (IQR) per species is shown. (DOCX) [file pone.0054784.s027.docx]

Supplementary Table S24: total decelerative impulse-- values are expressed in percentage bodyweight per second (%BW s); median impact impulse (IQR) per species is shown.

|  | **Forelimb Walk**  **Total Decelerative**  **Impulse (%BWs)** | | **Forelimb Slow Run**  **Total Decelerative**  **Impulse (%BWs)** | | **Hindlimb Walk**  **Total Decelerative**  **Impulse (%BWs)** | | **Hindlimb Slow Run**  **Total Decelerative**  **Impulse (%BWs)** | |
| --- | --- | --- | --- | --- | --- | --- | --- | --- |
|  |  |  |  |  |  |  |  |  |
|  |  |  |  |  |  |  |  |  |
| Antelope | 9.26 | (2.83) | 7.75 | (0.74) |  |  |  |  |
| Sheep | 3.35 | (2.75) | 4.06 | (3.74) | 4.16 | (0.87) | 3.00 | (1.77) |
| Pig | 1.46 | (0.47) | 1.47 | (0.19) | 1.23 | (0.35) | 1.18 | (1.59) |
| Addax | 5.76 | (1.97) |  |  | 1.72 | (0.96) |  |  |
| Alpaca |  |  |  |  |  |  |  |  |
| Deer | 1.64 | (0.67) | 2.07 | (0.65) | 0.50 | (0.37) | 0.92 | (0.18) |
| Horse | 2.61 | (2.18) | 4.13 | (1.52) | 1.84 | (1.55) | 2.37 | (0.09) |
| Bull | 3.08 | (1.90) |  |  | 2.67 | (1.02) |  |  |
| Dromedary | 1.87 | (0.39) |  |  | 1.17 | (0.19) | 0.79 | (0.01) |
| Giraffe | 6.88 | (1.47) |  |  |  |  |  |  |
| Elephant | 1.80 | (1.04) | 2.19 | (0.82) | 1.18 | (0.59) | 1.19 | (0.27) |
