# Supplementary material for: Size-Related Changes in Foot Impact Mechanics in Hoofed Mammals
Source: PLoS One. 2013 Jan 30;8(1):e54784. doi: 10.1371/journal.pone.0054784 (PMC3559824; doi:10.1371/journal.pone.0054784)
Supplement: Table S25 — Total accelerative impulse– MannWhitney U Test outcomes comparing limb and speed effects. (DOCX) [file pone.0054784.s028.docx]

Supplementary Table S25: total accelerative impulse-- MannWhitney U Test outcomes comparing limb and speed effects. * denotes significant differences between fore- and hind limbs, or between walk and slow run.

|  |  |  |  |  |  |
| --- | --- | --- | --- | --- | --- |
|  |  | **p value** | **Total N** | **Mann-Whitney U** | **Z** |
|  |  |  |  |  |  |
| Forelimb walk versus Hindlimb walk | Sheep | 0.480 | 25 | 65.0 | -0.707 |
|  | Pig | 0.091 | 35 | 101.0 | -1.689 |
|  | Addax | 0.001* | 17 | 3.0 | -3.175 |
|  | Deer | <0.001* | 48 | 16.0 | -5.603 |
|  | Horse | 0.059 | 56 | 277.0 | -1.885 |
|  | Bull | 0.088 | 44 | 169.0 | -1.704 |
|  | Dromedary | <0.001* | 32 | 10.0 | -4.355 |
|  | Elephant | 0.013* | 43 | 128.0 | -2.484 |
| Forelimb run versus Hindlimb run | Sheep | 0.302 | 9 | 5.0 | -1.033 |
|  | Pig | 0.336 | 17 | 26.0 | -0.962 |
|  | Deer | <0.001* | 20 | 6.0 | -3.240 |
|  | Horse | 0.001* | 14 | 0.0 | -3.003 |
|  | Elephant | 0.050 | 6 | 0.0 | -1.964 |
| Forelimb run versus Forelimb walk | Antelope | 0.176 | 24 | 16.0 | -1.353 |
|  | Sheep | 0.470 | 15 | 13.0 | -0.722 |
|  | Pig | 0.358 | 24 | 49.0 | -0.919 |
|  | Deer | 0.141 | 33 | 65.0 | -1.470 |
|  | Horse | 0.008* | 33 | 19.0 | -2.561 |
|  | Elephant | 0.602 | 26 | 28.0 | -0.602 |
| Hindlimb run versus Hindlimb walk | Sheep | 0.044 | 19 | 16.0 | -2.017 |
|  | Pig | 0.572 | 28 | 74.0 | -0.566 |
|  | Deer | 0.002* | 35 | 48.0 | -3.128 |
|  | Horse | 0.011* | 37 | 54.0 | -2.549 |
|  | Dromedary | 0.027 | 15 | 0.0 | -2.208 |
|  | Elephant | 0.784 | 23 | 27.0 | -0.274 |
