# Supplementary material for: Size-Related Changes in Foot Impact Mechanics in Hoofed Mammals
Source: PLoS One. 2013 Jan 30;8(1):e54784. doi: 10.1371/journal.pone.0054784 (PMC3559824; doi:10.1371/journal.pone.0054784)
Supplement: Table S26 — Total accelerative impulse– values are expressed in percentage bodyweight per second (%BW s); median impact impulse (IQR) per species is shown. (DOCX) [file pone.0054784.s029.docx]

Supplementary Table S26: total accelerative impulse-- values are expressed in percentage bodyweight per second (%BW s); median impact impulse (IQR) per species is shown.

|  | **Forelimb Walk**  **Total Accelerative**  **Impulse (%BWs)** | | **Forelimb Slow Run**  **Total Accelerative**  **Impulse (%BWs)** | | **Hindlimb Walk**  **Total Accelerative**  **Impulse (%BWs)** | | **Hindlimb Slow Run**  **Total Accelerative**  **Impulse (%BWs)** | |
| --- | --- | --- | --- | --- | --- | --- | --- | --- |
|  |  |  |  |  |  |  |  |  |
|  |  |  |  |  |  |  |  |  |
| Antelope | 1.88 | (1.89) | 6.49 | (1.28) |  |  |  |  |
| Sheep | 3.73 | (1.58) | 5.91 | (3.00) | 7.45 | (2.97) | 7.87 | (5.42) |
| Pig | 1.30 | (0.53) | 2.84 | (0.39) | 1.39 | (0.35) | 2.26 | (0.61) |
| Addax | 2.84 | (1.27) |  |  | 5.69 | (4.52) |  |  |
| Alpaca |  |  |  |  |  |  |  |  |
| Deer | 1.40 | (0.48) | 1.64 | (0.39) | 1.36 | (0.44) | 1.19 | (0.09) |
| Horse | 3.21 | (2.21) | 4.80 | (2.11) | 1.84 | (1.74) | 2.41 | (0.08) |
| Bull | 2.28 | (1.98) |  |  | 2.00 | (2.76) |  |  |
| Dromedary | 1.41 | (0.34) |  |  | 0.66 | (0.42) | 1.11 | (0.15) |
| Giraffe | 2.66 | (1.32) |  |  |  |  |  |  |
| Elephant | 3.88 | (2.80) | 4.52 | (0.76) | 0.99 | (1.11) | 1.36 | (1.25) |
