# Supplementary material for: Size-Related Changes in Foot Impact Mechanics in Hoofed Mammals
Source: PLoS One. 2013 Jan 30;8(1):e54784. doi: 10.1371/journal.pone.0054784 (PMC3559824; doi:10.1371/journal.pone.0054784)
Supplement: Table S27 — Ground speed matching (GSM): values represent ratio between the resultant centre of mass velocity and resultant foot velocity; median (IQR) per species is shown. (DOCX) [file pone.0054784.s030.docx]

Supplementary Table S27: ground speed matching (GSM): values represent ratio between the resultant centre of mass velocity and resultant foot velocity; median (IQR) per species is shown.

|  | **Forelimb Walk**  **GSM** | | **Forelimb Slow Run**  **GSM** | | **Hindlimb Walk**  **GSM** | | **Hindlimb Slow Run**  **GSM** | |
| --- | --- | --- | --- | --- | --- | --- | --- | --- |
|  |  |  |  |  |  |  |  |  |
|  |  |  |  |  |  |  |  |  |
| Antelope | 0.39 | (0.27) |  |  |  |  |  |  |
| Sheep | 0.88 | (0.21) |  |  | 0.98 | (0.06) | 0.78 | (0.02) |
| Pig | 0.87 | (0.24) | 0.72 | (0.29) | 0.73 | (0.54) | 0.79 | (0.14) |
| Addax |  |  |  |  |  |  |  |  |
| Alpaca | 0.70 | (0.25) | 0.41 | (0.28) | 0.80 | (0.34) | 0.68 | (0.13) |
| Deer | 0.13 | (0.25) | 0.53 | (0.10) | 0.15 | (0.06) | 0.39 | (0.22) |
| Horse | 0.84 | (0.14) | 0.85 | (0.29) | 0.68 | (0.38) | 0.75 | (0.09) |
| Bull | 0.54 | (0.17) |  |  | 0.44 | (0.16) |  |  |
| Dromedary | 0.63 | (0.15) |  |  | 0.64 | (0.20) |  |  |
| Giraffe | 0.48 | (0.18) |  |  |  |  |  |  |
| Elephant | 0.95 | (0.04) | 0.98 | (0.02) | 0.96 | (0.05) | 0.92 | (0.13) |
