# Supplementary material for: Size-Related Changes in Foot Impact Mechanics in Hoofed Mammals
Source: PLoS One. 2013 Jan 30;8(1):e54784. doi: 10.1371/journal.pone.0054784 (PMC3559824; doi:10.1371/journal.pone.0054784)
Supplement: Table S28 — Results of phylogenetically independent contrasts analysis. (DOCX) [file pone.0054784.s031.docx]

Supplementary Table S29: results of phylogenetically independent contrasts analysis.

| **log10(peak vertical impact force amplitude) vs. log10(M_b_)** | | | | | | | | | | | | | |
| --- | --- | --- | --- | --- | --- | --- | --- | --- | --- | --- | --- | --- | --- |
|  | OLS | | | | GLS | | | | OU | | | |  |
|  | *b* | *se* | *ML* | *AIC* | *b* | *se* | *ML* | *AIC* | *b* | *se* | *ML* | *AIC* | *d* |
| All = 1 | **0.686** | **0.083** | **3.63** | **-1.27** | 0.667 | 0.084 | 0.93 | 4.13 | 0.686 | 0.083 | 3.63 | 0.73 | 2.60E-17 |
| Pagels | Same AA |  |  |  | 0.588 | 0.071 | 1.83 | 2.34 | 0.686 | 0.083 | 3.63 | 0.73 | 2.60E-17 |
| **log10(M*_eff_*) vs. log10(M_b_)** | | | | | | | | | | | | | |
|  | OLS | | | | GLS | | | | OU | | | |  |
|  | *b* | *se* | *ML* | *AIC* | *b* | *se* | *ML* | *AIC* | *b* | *se* | *ML* | *AIC* | *d* |
| All = 1 | **0.849** | **0.140** | **-2.50** | **10.99** | 0.849 | 0.14 | -4.78 | 15.56 | 0.84900 | 0.14 | -2.50 | 12.99 | 2.15E-04 |
| Pagels | Same AA |  |  |  | 0.776 | 0.14 | -5.57 | 17.13 | 0.84900 | 0.14 | -2.50 | 12.99 | 2.60E-17 |
| **log10(peak vertical GRF amplitude) vs. log10(M_b_)** | | | | | | | | | | | | | |
|  | OLS | | | | GLS | | | | OU | | | |  |
|  | *b* | *se* | *ML* | *AIC* | *b* | *se* | *ML* | *AIC* | *b* | *se* | *ML* | *AIC* | *d* |
| All = 1 | **0.832** | **0.083** | **3.58** | **-1.17** | 0.838 | 0.073 | 2.46 | 1.09 | 0.8370 | 0.076 | 3.70 | 0.60 | 1.63E-01 |
| Pagels | Same AA |  |  |  | 0.787 | 0.071 | 1.88 | 2.25 | 0.826 | 0.077 | 3.77 | 0.46 | 5.78E-02 |
| **log10(vertical impact velocity) vs. log10(M_b_)** | | | | | | | | | | | | | |
|  | OLS | | | | GLS | | | | OU | | | |  |
|  | *b* | *se* | *ML* | *AIC* | *b* | *se* | *ML* | *AIC* | *b* | *se* | *ML* | *AIC* | *d* |
| All = 1 | **0.106** | **0.062** | **6.77** | **-7.54** | 0.08 | 0.060 | 4.61 | -3.23 | 0.106 | 0.063 | 6.77 | -5.54 | 2.60E-17 |
| Pagels | Same AA |  |  |  | 0.01 | 0.053 | 5.15 | -4.30 | 0.106 | 0.062 | 6.77 | -5.54 | 2.60E-17 |
| **log10(impact duration) vs. log10(M_b_)** | | | | | | | | | | | | | |
|  | OLS | | | | GLS | | | | OU | | | |  |
|  | *b* | *se* | *ML* | *AIC* | *b* | *se* | *ML* | *AIC* | *b* | *se* | *ML* | *AIC* | *d* |
| All = 1 | **0.073** | **0.076** | **4.49** | **-2.97** | 0.04 | 0.069 | 3.17 | -0.34 | 0.073 | 0.076 | 4.49 | -0.97 | 2.60E-17 |
| Pagels | Same AA |  |  |  | 0.03 | 0.068 | 2.43 | 1.14 | 0.073 | 0.076 | 4.49 | -0.97 | 2.60E-17 |
| **log10(loading rate (0.5% window during impact)) vs. log10(M_b_)** | | | | | | | | | | | | | |
|  | OLS | GLS | OU |  |  |  |  |  |  |  |  |  |  |
|  | *b* | *se* | *ML* | *AIC* | *b* | *se* | *ML* | *AIC* | *b* | *se* | *ML* | *AIC* | *d* |
| All = 1 | **0.641** | **0.059** | **7.40** | **-8.81** | 0.619 | 0.060 | 4.82 | -3.65 | 0.641 | 0.059 | 7.40 | -6.81 | 2.60E-17 |
| Pagel’s | Same AA |  |  |  | 0.572 | 0.058 | 3.47 | -0.950 | 0.641 | 0.059 | 7.40 | N/A | N/A |
