# Supplementary material for: Size-Related Changes in Foot Impact Mechanics in Hoofed Mammals
Source: PLoS One. 2013 Jan 30;8(1):e54784. doi: 10.1371/journal.pone.0054784 (PMC3559824; doi:10.1371/journal.pone.0054784)
Supplement: Methods S1 — Phylogenetically independent contrasts analysis. (DOCX) [file pone.0054784.s032.docx]

Supplementary Methods S28: Phylogenetically independent contrasts analysis.

We checked for bias caused by non-independence of species’ data points due to closeness of phylogenetic relationships in our dataset with phylogenetically independent contrasts (PIC) analysis [41-43]. For simplicity, we focused on logarithmically transformed data from the forelimb at walking speeds using six key parameters with the expectation that any presence/lack of phylogenetic bias here would apply similarly to other parameters.

We then built a character-taxon matrix for our data in Mesquite 2.7237, and constructed a cladogram (see Supplementary Figure S28) by consulting recent phylogenetic analyses of mammals, especially (cet)artiodactyls [44-47] *.* We first checked our data in the PDAP module of Mesquite [45] to ensure it was appropriate for analysis. Then we used REGRESSIONv243 code in Matlab R2012a software (Mathworks, Inc.; Natick, MA, USA) to consider differences between a simple ordinary least-squares regression (OLS; i.e., without PIC adjustments; a “star phylogeny”) and PIC analysis with different branch length assumptions (branch lengths set equal to 1 and using Pagel’s method [44]). The two PIC models used were generalized least squares (GLS) under an initial “speciational” model of branch lengths (all branches having lengths of 1) and a GLS analysis with branch lengths transformed following an Ornstein-Uhlenbeck model (OU). We compared the Akaike Information Criterion (AIC) values among these models (OLS, GLS, and OU; latter two using two branch length assumptions) to identify which best fit the data (lowest values of AIC).

We found that the OLS (non-phylogenetic) model consistently outperformed the phylogenetically corrected models, supporting our assumption that there is no clear phylogenetic bias in our scaling data. Even if there was a phylogenetic bias, the generally close agreement between the OLS and GLS/OU methods’ exponents intimates that it would not change our conclusions appreciably. To depict the scatter in the mean data for each of six parameters for each taxon’s forelimb walking speeds, we include Supplementary Figures S31 and S32. Our calculated exponents (Table S29) differed slightly from those in Tables 1 and 2 but not significantly.
