# Supplementary material for: Size-Related Changes in Foot Impact Mechanics in Hoofed Mammals
Source: PLoS One. 2013 Jan 30;8(1):e54784. doi: 10.1371/journal.pone.0054784 (PMC3559824; doi:10.1371/journal.pone.0054784)
Supplement: References S1 — (DOCX) [file pone.0054784.s033.docx]

Supplementary References S33:

41. Felsenstein J (1985) Phylogenies and the comparative method. Am Nat 125: 1-15.

42. Grafen A (1989) The phylogenetic regression. Phil Trans R Soc Lond B 326: 119-157.

43. Garland T, Harvey PH, Ives AR (1992) Procedures for the analysis of comparative data using phylogenetically independent contrasts. Syst Biol 41: 18-32.

44. Pagel MD (1992) A method for the analysis of comparative data. J Theor Biol 156: 431-442.

45. Maddison WP, Maddison DR (2009) Mesquite: a modular system for evolutionary analysis. Mesquite Version 2.72. http://mesquiteproject.org.

46. Hassanin A, Ropiquet A (2004) Molecular phylogeny of the tribe Bovini (Bovidae, Bovinae) and the taxonomic status of the Kouprey, Bossauveli Urbain 1937. Molec Phylogenet Evol 33: 896-907.

47. Bininda-Emonds OR, Cardillo M, Jones KE, MacPhee RDE, Beck RMD et al. (2007) The delayed rise of present-day mammals. Nature 446: 507-512.
